# Maternal-fetal attachment and interrelated factors in pregnant women assisted in Primary Health Care *

**DOI:** 10.1590/1518-8345.7104.4404

**Published:** 2024-10-25

**Authors:** Cássio de Almeida Lima, Maria Fernanda Santos Figueiredo Brito, Lucineia de Pinho, Sélen Jaqueline Souza Ruas, Romerson Brito Messias, Marise Fagundes Silveira

**Affiliations:** 1 Universidade Estadual de Montes Claros, Departamento de Métodos e Técnicas Educacionais, Montes Claros, MG, Brazil; ^2^ Scholarship holder at the Coordenação de Aperfeiçoamento de Pessoal de Nível Superior (CAPES), Brazil; ^3^ Universidade Estadual de Montes Claros, Departamento de Saúde Mental e Saúde Coletiva, Montes Claros, MG, Brazil; ^4^ Faculdade de Saúde e Humanidades Ibituruna, Departamento de Enfermagem, Montes Claros, MG, Brazil; ^5^ Universidade Estadual de Montes Claros, Departamento de Ciências Exatas, Montes Claros, MG, Brazil; ^6^ Scholarship holder at the Conselho Nacional de Desenvolvimento Científico e Tecnológico (CNPq), Brazil

**Keywords:** Pregnant Women, Maternal-Fetal Relations, Primary Health Care, Health Surveys, Multivariate Analysis, Community Health Nursing

## Abstract

to analyze maternal-fetal attachment and interrelated factors in pregnant women assisted in Primary Health Care.

a cross-sectional, population-based, analytical epidemiological survey. A sample of 937 participants attended by Family Health Strategy teams was investigated. Maternal-fetal attachment (outcome), sociodemographic and clinical variables, social support, family functionality, depressive symptoms and perceived stress were assessed. Multivariate analysis was adopted using structural equation modeling.

maternal-fetal attachment had an average of 92.6 (SD=±15.3). The adjusted structural model showed that the following factors had a direct effect on the outcome: gestational weeks (β=0.29; p<0.001), household crowding (β=-0.07; p=0.027), depressive symptoms (β=-0.11; p=0.003), social support (β=0.08; p<0.001) and family functionality (β=0.19; p<0.001). Indirect effects of social support (β=-0.29; p<0.001) and family functionality (β=-0.20; p<0.001) were identified, mediated by depressive symptoms.

a set of interrelationships was identified between maternal-fetal attachment, gestational weeks, household crowding, depressive symptoms, social support and family functionality. It is suggested that the Family Health Strategy offer prenatal care anchored in integrality and humanization, which promotes biopsychosocial well-being during pregnancy and healthy maternal-fetal attachment.

## Highlights

**(1)** Maternal-fetal attachment should be assessed in Primary Health Care.

**(2)** Depressive symptoms were negatively related to maternal-fetal attachment.

**(3)** Social support and family functionality had a positive effect on attachment.

**(4)** Greater household crowding had a negative effect on the outcome.

**(5)** It is recommended to screen pregnant women with depression, poor social and family support.

## Introduction

 The pregnancy cycle is considered a transitional period that is part of the development process, involving a change in identity and a redefinition of roles in a woman’s life ^(^ 1 ^-^ 2 ^)^ , as well as hormonal and emotional changes ^(^ 3 ^)^ . It is characterized as a phase permeated by adaptations to biopsychosocial changes, in which women have to reconcile the demands and challenges of motherhood with the family, marital and professional roles they play socially ^(^ 1 ^-^ 2 ^)^ . 

 The successful adaptation of the pregnant woman to the process of transition to motherhood is closely linked to the development of the affective bond with the fetus, i.e. the mental representation of the fetus and the feelings of being attached to it ^(^ 4 ^-^ 5 ^)^ . It is in the prenatal phase that the mother’s relationship with her child begins: maternal-fetal attachment (MFA), which involves the intensity of affiliation and interaction behaviors with the unborn child. The pregnant woman tends to establish an emotional connection with the fetus and experiences expectations regarding the child to come, which increase throughout pregnancy ^(^ 3 ^-^ 5 ^)^ . MFA involves maternal expectations, emotions, thoughts and behaviours in relation to the fetus, pregnancy and motherhood, and is an important predictor of the mother-baby bond in the postpartum period ^(^ 2 ^,^ 4 ^-^ 5 ^)^ . Attachment can be a predictive factor of maternal puerperal mental state, gestational self-care, neonatal outcomes, child development and behavioral and socio-emotional skills ^(^ 5 ^)^ . It is a multidimensional construct ^(^ 2 ^-^ 14 ^)^ , permeated by sociodemographic questions ^(^ 3 ^,^ 7 ^-^ 8 ^,^ 13 ^-^ 14 ^)^ , obstetrics ^(^ 3 ^,^ 7 ^-^ 9 ^,^ 13 ^-^ 14 ^)^ , cultural ^(^ 2 ^,^ 13 ^-^ 14 ^)^ and psychological ^(^ 2 ^-^ 14 ^)^ . 

 The investigation of MFA by nurses in prenatal care in the public health system can contribute to screening pregnant women at risk of developing poor attachment, who need a multi-professional approach with a view to the quality of the mother-fetus bond and healthy family relationships. It is necessary to provide holistic care to pregnant women who use the Unified Health System (SUS) and Primary Health Care (PHC), which also includes assessing MFA. The examination of this event should consider interfering aspects in specific sociocultural environments ^(^ 13 ^-^ 15 ^)^ , as in communities covered by the Family Health Strategy (FHS). In Brazil, these women live in a context that is at odds with previous studies in the international literature ^(^ 4 ^-^ 5 ^,^ 7 ^-^ 12 ^)^ . 

 It is also necessary to consider the national guidelines that lead the maternal and child care line and prenatal care in Brazil ^(^ 2023 ^)^ . The National Policy for Compreh 

 ensive Women’s Health Care (PNAISM) incorporates, however a gender and the empowerment of SUS users bias, integrality and health promotion as its guiding principles, aspiring to consolidate advances in the field of sexual and reproductive rights ^(^ 17 ^)^ . The Stork Network ( *Rede Cegonha* ) consists of a network of care aimed at reproductive planning and humanized care for women during pregnancy, childbirth and the puerperium. It aims to ensure the safe birth, healthy growth and development of children ^(^ 18 ^)^ . 

 Evidence on MFA is incipient in PHC, especially in Latin American countries and Brazil, where population-based epidemiological research is insufficient ^(^ 2 ^,^ 14 ^)^ carried out with pregnant women attending FHS services. Based on broad epidemiological surveys, it is possible to suggest improvements in prenatal care, with a view to promoting healthy MFA ^(^ 15 ^)^ , this makes it necessary to carry out national research that simultaneously assesses MFA and its determinants in pregnant women who use PHC, where most of them are seen. The other studies ^(^ 2 ^-^ 14 ^)^ used common inferential statistical techniques, which indicates the need for research with more robust analyses, such as structural equation modeling (SEM). Therefore, the aim of this study was to analyze MFA and interrelated factors in pregnant women assisted in PHC. 

## Method

### Study design

 This is an observational epidemiological survey with a sectional design, population-based and analytical design, which used data from a large study entitled “Evaluation of the health conditions of pregnant women in Montes Claros - MG: longitudinal study (ALGE Study)”. The recommendations of Strengthening the Reporting of Observational Studies in Epidemiology (STROBE) ^(^ 19 ^)^ were followed. 

### Scenario

 The setting for the study was Montes Claros city, located in the northern region of the state of Minas Gerais (MG), Brazil. This city is considered a regional hub and has an estimated population of 417,478 inhabitants. The local FHS services were set up in the 1990s and are currently organized into 15 poles. These centers had a total of 135 family health teams at the time of the investigation (2018-2020), covering 100% of the population ^(^ 20 ^)^ . 

### Sample size

The population of the ALGE Study was made up of pregnant women registered with the FHS teams in the urban area of the municipality in 2018-2020. The sample size was established in order to estimate population parameters with a prevalence of 50% (to maximize the sample size and because the original project included several outcomes). A 95% confidence interval (95% CI) and precision level of 2.0% were considered. Correction was made for a finite population (N=1,661) and an additional 20% was included to compensate for possible non-response and losses. The calculations showed that at least 1,180 pregnant women needed to take part.

To select the sample, the FHS centers in all regions of the municipality were taken into account, which totaled 15 at the time of this study and among which 135 family health teams were distributed. The number of pregnant women sampled in each center was proportional to their representativeness in relation to the total population of registered pregnant women.

Pregnant women who were registered with a family health team at any gestational age were included. Women who were pregnant with twins (as this could affect certain variables measured in the project) and those with cognitive impairment, according to a previous medical diagnosis informed by the family member and/or the FHS team, were not included.

 This study only analyzed data from women in the second and third trimesters of pregnancy. This was because MFA, as measured by the Maternal-Fetal Attachment Scale (MFAS), is more evident from the second trimester onwards. As the fetus grows, the pregnant woman can feel the baby’s new movements, which makes the experience more corporeal for her and allows for a more vivid interaction with the fetus ^(^ 4 ^,^ 13 ^)^ . In this sense, this study only considered the population of pregnant women in the 2 ^nd^ and 3 ^rd^ trimesters of pregnancy (N=1,218), so the minimum sample size was estimated at n=930 pregnant women. The same parameters adopted for calculating the sample size of the ALGE Study were maintained. In addition, the sample size defined for this investigation met the premises of the statistical treatment of data adopted in SEM, in which it is recommended that the sample have at least 250-500 observations ^(^ 21 ^)^ . 

### Instruments and variables

To characterize the participants in this study, a structured questionnaire was used which included sociodemographic variables - age group (up to 20 years old, 21 to 30, over 30), marital status (with partner, without partner), self-declared color (brown, black, white, yellow), schooling (elementary, high school, college), monthly family income (less than R$ 1.000, R$ 1,001 to 2,000, more than R$ 2,000); clinical variables - gestational trimester (second trimester of pregnancy), number of residents in the household, number of rooms in the household. 000.00, R$ 1,001.00 to 2,000.00, more than R$ 2,000.00), number of residents in the household, number of rooms in the household; and clinical - gestational trimester (second, third), pregnancy planning (yes, no), previous abortion (yes, no), weeks of gestation. The sociodemographic variable household agglomeration was calculated by the ratio “number of residents in the household/number of rooms in the household”. The gestational weeks and household agglomeration variables (numerical and observed) were included in the structural model.

Nationally validated instruments were also used to investigate the following constructs: MFA, depressive symptoms, perceived stress, social support and family functionality.

 The Brazilian version of the EAMF ^(^ 22 ^)^ was applied to assess the main outcome of this survey, MFA. MFA was treated as an observed, discrete numerical variable. The EAMF was developed by Nurse Mecca S. Cranley in 1981 in the United States of America (USA) ^(^ 23 ^)^ . It contains 24 items answered on a Likert scale from one to five points (never to almost always). The minimum score is 24 and the maximum is 120. In the validation process of the EAMF for the Brazilian population, the unidimensionality of the construct was observed and its use was recommended based on the total score. Thus, higher scores indicate higher levels of AMF ^(^ 22 ^)^ . The following classification is proposed: low level of attachment (24 to 47 points), medium (48 to 97) and high (98 to 120) ^(^ 24 ^)^ . Cronbach’s α coefficient was 0.874 in this study. 

 The US Center for Epidemiologic Studies Depression Scale (CES-D), also validated in Brazil, was used as a population screening scale for depression ^(^ 25 ^)^ , was used to screen for depressive symptoms in the sample of pregnant women evaluated. The CES-D consists of 20 items, four of which are positive, in which the interviewee reports the frequency of occurrence of symptoms in the last week. Each answer can involve four increasing degrees of intensity on a Likert scale - never or rarely, sometimes, often and always - with scores corresponding to 0, 1, 2 and 3. The score of the four positive items is inverted and added to the score of the others, giving a final result ranging from zero to 60 - the higher the score, the greater the intensity of symptoms. It was categorized into: absent/mild depressive symptoms (score <16), moderate (score ≥16 or ≤21) and severe symptoms (score ≥22) ^(^ 25 ^-^ 26 ^)^ . The instrument had a satisfactory Cronbach’s α (0.777). 

 Perceived stress was assessed using the Perceived Stress Scale (PSS-14), validated in Brazil. The instrument identifies situations in the individual’s life that are considered stressful, establishing levels of intensity. The questions are of a general nature and apply to any population subgroup. It contains 14 items about the frequency with which certain feelings and thoughts have occurred in the last month, with answers ranging from zero (never) to four (always). The score is obtained by reversing the scores of the positive items and adding up the answers to the 14 items, with a total score ranging from zero (no stress symptoms) to 56 (extreme stress symptoms). The variable was dichotomized into absence of stress (scores less than or equal to 30) and presence of stress (above 30) ^(^ 27 ^)^ . Cronbach’s α=0.782 was found for the PSS-14 in this study, an appropriate value. 

 The presence of social support was measured using the Brazilian version of the Social Support Scale, which was also validated in Brazil. The scale is made up of 19 questions comprising five dimensions: material, affective, emotional, positive social interaction and information. For each item, the participant indicates how often they consider each type of support, using a Likert scale: never (1), rarely (2), sometimes (3), almost always (4) and always (5). The closer the final score is to 100, the better the perceived social support. The overall score of the scale was calculated from the total sum of the 19 items and a result higher than 66, which corresponds to the second tertile, was considered high social support ^(^ 28 ^)^ . The instrument showed high internal consistency, with a Cronbach’s **α** of 0.960. 

 A nationally validated instrument called APGAR Familiar was used to assess family functionality ^(^ 29 ^)^ . It signals compliance with basic parameters defined by the acronym APGAR: A - Adaptation; P - Participation; G - Growth; A - Affection; R - Resolution. The questionnaire has five questions with three possible answers each and scores ranging from zero to two points - always (2), sometimes (1) and never (0). This gives a total score of between zero and ten points, which, the higher the score, indicates that the participant is more satisfied with their family. They were categorized into functional families (scores of 7-10) and dysfunctional families (<6) ^(^ 30 ^)^ . The instrument obtained adequate reliability in this investigation (Cronbach’s α=0.872). 

The variables MFA, depressive symptoms, perceived stress, social support and family functionality were also analyzed as observed and numerical, through their respective scores.

### Data collection

As for the data collection process, the managers of the municipality’s PHC coordination were initially contacted to raise awareness and explain the purpose of the research. Once they had agreed, the researchers also visited the family health teams to clarify the study. The professionals in these teams responsible for prenatal care provided a list of pregnant women in their area of coverage, containing their names and addresses. Once they had these lists, a team of interviewers made initial contact with the women, inviting them to come in and make them aware of the study, and then scheduled and carried out the data collection.

The data was collected by a multi-professional health team and undergraduate students (Physical Education, Nursing and Medicine courses) between October 2018 and February 2020, at the FHS health units or at the participants’ homes, depending on their availability. Data collection took place face-to-face, individually with each pregnant woman, lasting an average of one hour. As for the order in which the instruments were applied, first a structured questionnaire was applied to investigate sociodemographic/economic and clinical characteristics, followed by scales relating to MFA, social support, family functionality, perceived stress and depressive symptoms.

Prior to data collection, the interviewers were trained, and a pilot study was carried out with 36 pregnant women registered at an FHS unit (who were not included in the study’s analysis), in order to standardize the data collection procedures.

### Data processing and analysis

Multivariate analysis was used using SEM. First, a hypothetical model was developed in order to assess the interrelationships between MFA, considered the main outcome, and the other variables investigated: gestational weeks, household crowding, symptoms of depression, perceived stress, social support and family functionality.

 According to the hypothesized model, gestational age (weeks of gestation) was placed in a position of direct correlation with the main outcome ^(^ 7 ^-^ 8 ^,^ 13 ^-^ 15 ^)^ . It has been theorized that household agglomeration also has a direct effect on MFA, as it is a possible marker of the pregnant woman’s socioeconomic status ^(^ 4 ^,^ 6 ^,^ 15 ^)^ . Perceived stress is also related to MFA ^(^ 5 ^,^ 7 ^,^ 9 ^,^ 15 ^)^ , as are depressive symptoms ^(^ 4 ^-^ 5 ^,^ 7 ^,^ 9 ^-^ 11 ^,^ 14 ^)^ . Social support has direct and indirect effects on MFA, in an interrelationship mediated by perceived stress and symptoms of depression ^(^ 4 ^-^ 6 ^,^ 12 ^,^ 15 ^)^ . The same assumption was made for the family functionality construct ^(^ 7 ^,^ 14 ^)^ . 

**Figure 1:**
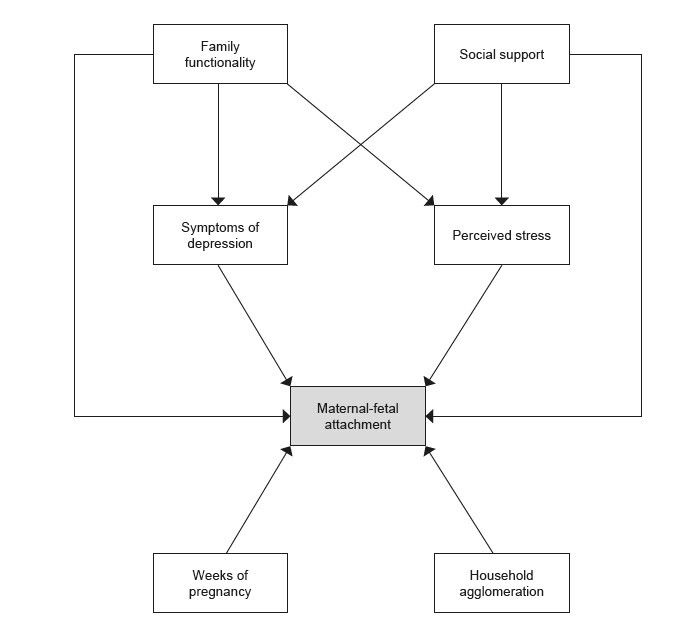
- Hypothetical model to analyze the factors interrelated to maternal-fetal attachment in pregnant women assisted in Primary Health Care. Montes Claros, MG, Brazil, 2018-2020

Figure 1 illustrates the direct and indirect relationships between the variables investigated in the proposed model. The observed variables are represented by rectangles and the correlations are indicated by arrows (from the independent variable to the dependent variable) ^(^ 31 ^)^ . 

 In the statistical analysis of the data, the categorical variables were initially described using their frequency distributions. Numerical variables were described using the mean, standard deviation (SD), minimum and maximum values, asymmetry (sk) and kurtosis (ku) coefficients. Values of sk> 3 and ku> 10 were accepted as indicators of a violation of the normality assumption ^(^ 31 ^)^ . Missing values were imputed by the mean. 

 The multivariate model was then adjusted using SEM. Direct and indirect effects were estimated, represented by standardized coefficients (SCs), whose statistical significance was assessed by the critical ratio (CR) at the 5% level. The standardized PCs were interpreted as follows: small effect when the values were close to 0.10, medium when close to 0.30 and large if greater than 0.50 ^(^ 32 ^)^ . The indirect effects, mediated by intermediate variables, were calculated by multiplying the coefficients of the model’s indirect paths. 

 The following indices were used to assess the adequacy of the overall fit of the model: chi-squared ratio by degrees of freedom (x ^2^ /gl), confirmatory fit index (CFI), goodness of fit index (GFI), Tucker-Lewis index (TLI) and root mean square error of approximation (RMSEA). The model fit was considered satisfactory if x ^2^ /df ≤ 5.0; CFI, GFI and TLI ≥ 0.90; RMSEA < 0.10 ^(^ 33 ^)^ . 

The data was organized and statistically analyzed using the IBM Statistical Package for the Social Sciences (SPSS) Statistics software, version 23.0. The data collected was previously subjected to quality control and double-checking. The structural modeling was processed in the Analysis of Moment Structures software (IBM SPSS Amos 23.0).

### Ethical aspects

This study complied with international and national ethical regulations for research involving human beings. The project was approved by the Research Ethics Committee Involving Human Beings of the State University of Montes Claros (CAAE 80957817.5.0000.5146, Consubstantiated Opinion number 2.483.623/2018 and Consubstantiated Opinion number 3.724.531/2019) and obtained institutional agreement from the Municipal Health Department. Participants of legal age read and signed the Free and Informed Consent Form. Those under the age of 18 presented the Free and Informed Consent Form, in addition to the Free and Informed Consent Form signed by their legal guardians.

## Results

**Figure 2:**
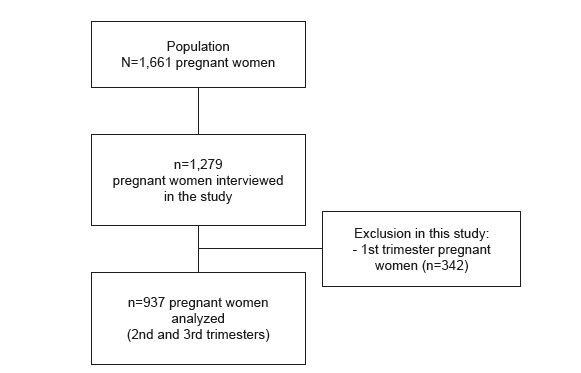
- Flowchart of the selection process for the sample of pregnant women analyzed in the study. Montes Claros, MG, Brazil, 2018-2020

 A total of 1,279 pregnant women took part in the baseline study. This study included data from 937 interviewees who were in the second and third trimesters of pregnancy ( Figure 2 ). 

 As for the sociodemographic and clinical characteristics of the participants in the sample analyzed (n=937), 47.7% were aged between 21 and 30, 77.2% reported being married to a partner, 46.7% had a monthly family income of up to 1,000 reais. Of those interviewed, 55.0% were in their second trimester, 61.0% reported an unplanned pregnancy, 48.7% were nulliparous and 18.9% reported a previous abortion. With regard to the MFA scores, there was an average of 92.6 (SD=±15.3) and the medium attachment level was the predominant one, found in 575 (61.4%) pregnant women. The deviations from normality were not very severe for the asymmetry and kurtosis coefficients of the variables in the structural model ( Table 1 ). 

**Table 1:** - Characteristics of pregnant women assisted in Primary Health Care and descriptive measures of the structural model variables (n = 937). Montes Claros, MG, Brazil, 2018-2020

Variables		n*	% [95%CI ^†^ ]	
Age group (years)				
Up to 20		203	22.4 [19.2-24.4]	
21 to 30		453	47.6 [45.3-51.6]	
Above 30		281	30.0 [27.1-33.0]	
Marital status				
With a partner		721	77.2 [74.4-79.8]	
Without a partner		213	22.8 [20.2-25.6]	
Self-declared color				
Brown		641	68.8 [65.7-71.7]	
Black		147	15.8 [13.6-18.3]	
White		102	10.9 [9.1-13.1]	
Yellow		42	4.5 [3.4-6.0]	
Education				
Elementary school		141	15.1 [12.8-17.4]	
High school		595	63.5 [60.4-55.6]	
Higher education		200	21.4 [18.8-24.0]	
Monthly family income (R$‡)				
Up to 1,000.00		422	46.7 [43.4-50.0]	
1.000,00 a 2.000,00		280	31.0 [28.0-34.0]	
Above 2.000,00		202	22.3 [19.6-25.0]	
Gestational trimester				
2 ^nd^		515	55.0 [51.8-58.2]	
3 ^rd^		422	45.0 [41.8-48.2]	
Pregnancy planning				
Yes		360	39.0 [359-42.1]	
No		564	61.0 [57.9-64.1]	
Previous abortion				
No		742	81.1 [78.6-83.6]	
Yes		173	18.9 [16.4-21.4]	
Maternal-fetal attachment				
Low		10	1.1 [0.4-1.8]	
Medium		575	61.4 [59.3-64.5]	
High		352	37.5 [34.4-40.6]	
Family functionality				
Functional family		785	84.0 [81.6-86.4]	
Dysfunctional family		149	16.0 [13.6-18.4]	
Social support				
Low		166	17.9 [15.4-20.4]	
High		760	82.1 [79.6-84.6]	
Depressive symptoms				
Absent/mild		560	61.3 [58.1-64.5]	
Moderate		142	15.5 [13.2-17.8]	
Severe		212	23.2 [20.5-25.9]	
Perceived stress				
Absence of stress		768	83.1 [80.7-85.5]	
Presence of stress		156	16.9 [14.5-18.3]	
Structural model variables	Mean(SD§)	Min-Max||	Sk *¶*	Ku**
Maternal-fetal attachment	92.6 (15.3)	29.0-120.0	-0.71	1.03
Family functionality	8.5 (2.1)	0.0-10.0	-1.72	2.68
Social support	79.3 (18.3)	0.0-94.7	-1.39	1.52
Depressive symptoms	15.3 (10.1)	0.0-57.0	1.17	1.22
Perceived stress	23.5 (8.7)	1.0-56.0	0.15	0.57
Gestational weeks	26.3 (7.6)	13.6-45.1	0.11	-0.95
Household agglomeration	0.7 (0.3)	0.20-3.50	2.22	8.31

* n varies due to missing information

^†^ 95%CI = 95% confidence interval

^‡^ R$ = Brazilian Real (minimum wage: R$ 954.00)

^§^ SD = Standard deviation

^||^ Min-Max = Minimum and maximum

^¶^ sk = Asymmetry

** ku = Kurtosis

**Figure 3:**
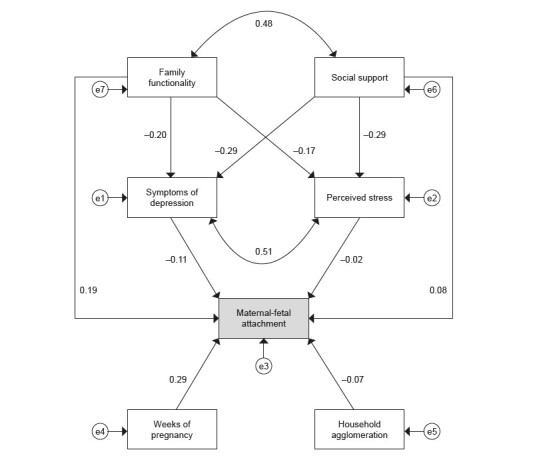
- Adjusted structural model of factors interrelated to maternal-fetal attachment in pregnant women assisted in Primary Health Care (n = 937). Montes Claros, MG, Brazil, 2018-2020

Figure 3 shows the interrelationships between the factors investigated and the standardized structural coefficients. The adjusted structural model obtained adequate quality of fit indicators, with the following values: x ^2^ /gl=2.29, GFI=0.994, CFI=0.989, TLI=0.975, RMSEA=0.037 (90% CI=0.016; 0.059, p-value=0.823). According to the model, weeks of gestation had a positive direct effect on the MFA outcome (β=0.29; p<0.001), while household agglomeration had a negative direct effect (β=-0.07; p=0.027). Depressive symptoms had a negative direct effect on the outcome (β=-0.11; p=0.003). Social support showed a positive and direct correlation with attachment (β=0.08; p<0.001), as well as an indirect negative effect mediated by the depressive symptoms variable (β=-0.29; p<0.001). As for family functionality, there was also a direct positive effect on MFA (β=0.19; p<0.001), as well as a negative effect in interrelation with depressive symptoms (β=-0.20; p<0.001). 

 The magnitudes of the direct, indirect and total effects are described in Table 2 . In the mediation analysis, only perceived stress did not obtain statistical evidence of a relationship with the event analyzed. 

**Table 2:** - Magnitudes of the estimated direct, indirect and total effects of factors interrelated to maternal-fetal attachment in pregnant women assisted in Primary Health Care (n = 937). Montes Claros, MG, Brazil, 2018-2020

Independent variable	Effect	Dependent variable	Standardized coefficient	Total	p-value*
	Direct/Indirect		Direct/Indirect		
Gestational weeks	Direct	Maternal-fetal attachment	0.29	0.29	<0.001
Household agglomeration	Direct	Maternal-fetal attachment	-0.07	-0.07	0.027
Depressive symptoms	Direct	Maternal-fetal attachment	-0.11	-0.11	0.003
Perceived stress	Direct	Maternal-fetal attachment	-0.02	-0.02	0.539
Social support	Direct	Maternal-fetal attachment	0.08		
	Indirect via depressive symptoms		-0.29x(-0.11)=0.0319	0.12	<0.001
	Indirect via perceived stress		-0.29x(-0.02)=0.0058		
	Direct	Depressive symptoms	-0.29	-0.29	<0.001
	Direct	Perceived stress	-0.29	-0.29	<0.001
Family functionality	Direct	Maternal-fetal attachment	0.19		
	Indirect via depressive symptoms		-0.20x(-0.11)=0.022	0.22	<0.001
	Indirect via perceived stress		-0.17x(-0.02)=0.0034		
	Direct	Depressive symptoms	-0.20	-0.20	<0.001
	Direct	Perceived stress	-0.17	-0.17	<0.001

* Statistical significance level (p<0.05)

## Discussion

This study showed the interrelationships between MFA, gestational age, household crowding, depressive symptoms, social support and family functionality in Brazilian pregnant women assisted in PHC. Using SEM, it was possible to understand a system made up of multiple factors and their interrelationships.

 In the adjusted structural model, it was observed that the weeks of gestation had a direct relationship with the event studied, proving to be a positive result. A similar result has been identified in research with Italian pregnant women ^(^ 8 ^)^ and Brazilians in the South of the country ^(^ 13 ^-^ 14 ^)^ . This is a plausible finding, since MFA behaviors increase with advancing gestational age as the woman feels the fetal movements, which makes the experience more bodily for her and accentuates the mother-baby interaction. From the fourth month of pregnancy onwards, with the movements of the fetus, the mother begins to express her representations, expectations, concerns and affections towards her unborn child more intensely ^(^ 8 ^,^ 13 ^-^ 14 ^)^ . 

 On the other hand, household crowding showed a negative relationship with MFA in the pregnant women interviewed: there was a downward trend in attachment scores as the number of people in the household increased. This factor can be considered a marker of the socioeconomic status of the mother and her family, which compromises the quality of the mother-infant emotional bond. Positive, stable and stimulating home environments enable the mother-infant relationship to be established in a healthier way. When this situation is not present, it can negatively influence the quality of maternal interaction, as it is a source of family stress ^(^ 6 ^)^ . Low socioeconomic conditions and unfavorable living contexts can hinder maternal and family adaptation to pregnancy, since pregnancy requires restructuring in several dimensions ^(^ 3 ^)^ . 

 Another negative aspect of this epidemiological survey was the presence of depressive symptoms, which were negatively correlated with MFA. This finding has been evidenced in international literature, in countries such as Denmark ^(^ 4 ^,^ 34 ^)^ , China ^(^ 5 ^)^ , Iran ^(^ 11 ^)^ , Turkey ^(^ 9 ^)^ and Portugal ^(^ 10 ^)^ ; as well as in a systematic review ^(^ 7 ^)^ . In Brazil, it was also observed in a household-based study in the South region ^(^ 14 ^)^ . Pregnancy may not be a protective factor for maternal mental health; on the contrary, it may place women in a position of vulnerability to psychological distress ^(^ 1 ^)^ . The multiple changes experienced during pregnancy require adaptations and can favor the development of psychopathologies ^(^ 3 ^)^ , such as depression, which is common at this stage ^(^ 7 ^)^ . This condition can interfere with the affective bond between the pregnant woman and the fetus during prenatal care ^(^ 3 ^,^ 14 ^,^ 34 ^)^ . It is theorized that depression affects the maternal empathic capacity and availability of affection, so the pregnant woman starts to consider the fetus as a source of irritation or guilt, resulting in a state of detachment ^(^ 14 ^,^ 34 ^)^ . However, it is not possible to attest to a direct causal relationship between depression and MFA, given the subjective and unique nature of these conditions, which requires caution when interpreting the observed results. Qualitative and longitudinal studies could help to deepen our understanding of this finding. 

 Pregnant women with depressive symptoms tend to experience motherhood as gloomy and permeated by feelings of guilt, sadness, fatigue, low self-esteem and helplessness. There may be a lack of maternal confidence in carrying out daily activities and caring for the future baby as desired, as well as fear, tension and intrusive thoughts of threats to the child’s life or well-being. It is a situation that generates suffering and anguish for the woman, who may even hide her mental state for fear of being stigmatized. There is a negative impact on maternal and child health, since depressive symptoms can continue into the puerperium ^(^ 5 ^,^ 15 ^,^ 35 ^)^ . Children of depressed mothers may have impaired affective, social and cognitive development ^(^ 1 ^)^ . Therefore, in the context of nursing care in the FHS, early identification of women in psychological vulnerability is recommended ^(^ 15 ^)^ , as well as attention to the mental health of pregnant women during prenatal care, in order to prevent depression ^(^ 6 ^)^ . Humanized care is also necessary, as recommended by the Brazilian Ministry of Health in its official guidelines ^(^ 17 ^-^ 18 ^,^ 36 ^-^ 37 ^)^ . 

 Expanded, resolutive and humanized professional care is also important when you consider that, in this investigation, there was statistical evidence of a positive and direct relationship between social support and attachment: the higher the level of social support, the greater the intensity of MFA. There was a negative indirect effect mediated by the depressive symptoms variable in the interrelationship between social support and the main outcome, i.e. the greater the social support of the pregnant woman, the lower the intensity of depressive symptoms, which had a negative impact on the mother-infant emotional bond. The connection between social support and MFA was also recorded in studies of Danish pregnant women ^(^ 4 ^)^ , Iranians ^(^ 11 ^)^ and Turkish ^(^ 9 ^)^ . High social support can contribute to the well-being of the mother-fetus binomial, since pregnant women who have a network of support and care are presumably more likely to take care of themselves and bond with the fetus ^(^ 14 ^)^ . This construct is important for maintaining mental health, coping with stressful situations and adapting the maternal behaviors required in the pregnancy-puerperal phase. Good social support can be an aspect that protects against the occurrence of depressive symptoms and promotes healthy motherhood. The role of this support is essential for buffering the stressful factors that occur in everyday life, especially in the case of pregnancy, when a number of psychosocial and physiological changes take place ^(^ 6 ^,^ 38 ^)^ . 

 Regarding family functionality, this research showed a beneficial result similar to social support. A direct positive effect on MFA was observed, as well as an effect mediated by depressive symptoms. It was inferred that adequate family functionality implies better MFA and lower intensity of depressive symptoms, which shows the favorable repercussions of a healthy family network on the event under analysis. During pregnancy, women need family support in order to adapt to the new conditions arising from the transition to motherhood ^(^ 11 ^)^ . This foundation, especially at times of vulnerability in the life cycle, has been shown to be a protective factor for maternal mental health ^(^ 1 ^,^ 14 ^)^ . A satisfactory family relationship reveals a greater perception of practical and emotional support, fosters feelings of belonging and enhances the performance of the maternal role ^(^ 1 ^)^ . This contributes directly to a life context that is more conducive to the establishment of MFA. In the community setting, it is recommended that nurses and FHS teams responsible for prenatal care take an approach aimed at promoting the psychosocial well-being and healthy family relationships of pregnant women ^(^ 15 ^)^ , as stipulated in the National Primary Care Policy (PNAB) ^(^ 36 ^)^ and the recommendations for low-risk prenatal care ^(^ 37 ^)^ . 

 In view of the findings identified in this study, there is a body of international ^(^ 4 ^-^ 5 ^,^ 9 ^,^ 11 ^,^ 34 ^)^ and Brazilian ^(^ 2 ^-^ 3 ^,^ 13 ^-^ 14 ^,^ 35 ^,^ 38 ^)^ evidence that can guide the practice of family health nurses. This evidence guides the screening of vulnerable pregnant users of PHC services, with symptoms of depression, difficulty adapting to pregnancy, little social and family support, who require care aimed at promoting mental health. There is a need for greater psychosocial and emotional support for pregnant women through a more holistic approach by the FHS teams, which can have a positive influence on MHFA ^(^ 5 ^,^ 9 ^,^ 15 ^,^ 35 ^,^ 39 ^)^ . In addition to the physical aspects, the subjective dimension that permeates the gestational period, such as MFA, should be considered by nurses and other family health professionals, as it is associated with better maternal and child outcomes in the postpartum period. These professionals need to assess attachment and encourage it early on. Health education groups with pregnant women during prenatal care offered at FHS units can be beneficial, as they allow participants to share their fears, anxieties and expectations, providing a sense of support ^(^ 2 ^,^ 39 ^)^ . 

 With a view to promoting humanized, resolutive and comprehensive care, as well as the quality of life of the maternal-fetal binomial, it is recommended that FHS team nurses base their prenatal care on the Ministry of Health’s national guidelines for maternal and childcare. These include the PNAISM ^(^ 17 ^)^ , the *Rede Cegonha* ^(^ 18 ^)^ , the PNAB ^(^ 36 ^)^ and the guidelines in the *Cadernos de Atenção Básica* document on professional practice in low-risk prenatal care ^(^ 37 ^)^ . In everyday health care, another suggestion is to apply the MFS in prenatal consultations and groups, in order to identify pregnant women who have difficulty establishing an emotional bond with the fetus. In the context of the FHS and the SUS, it is possible to screen pregnant women and families who need a professional approach at an early stage, with a view to improving the quality of the mother-fetus bond ^(^ 15 ^)^ . 

This study has certain limitations. The information was self-reported by the participants and is therefore prone to social acceptability bias and memory bias. Although the excess of variables in the model may hinder the quality of the fit, other variables, mainly relating to socioeconomic profile, religion and lifestyle were not analyzed. It is suggested that further studies be carried out with a prospective longitudinal design to examine the causal relationship between the factors verified and MFA, with the addition of these variables. Another recommendation is to include pregnant women from rural areas in future studies.

Despite these limitations, it is worth highlighting the positive aspects of this investigation. It was a large population-based epidemiological survey, with a significant sample and covering the entire urban area covered by the FHS teams. Instruments validated in Brazil were used, making the results more reliable. The analysis with SEM provided greater robustness and consistency to the research. The findings obtained can contribute to adding epidemiological evidence on the subject in the context of PHC, especially when recognizing the national novelty of this survey, as it involves pregnant women assisted by family health teams.

## Conclusion

This study showed a network of interrelationships between predictors of MFA in pregnant women assisted by FHS teams. The adjusted structural model revealed that higher gestational age, greater social support and better family functionality had a positive relationship with MFA. Greater household crowding and depressive symptoms had a negative impact on this construct. There was a protective role for adequate social and family support in the mental health of pregnant women. These results are important for PHC nurses who provide prenatal care, as they indicate the need for comprehensive and humanized care that promotes biopsychosocial well-being, mental health, prevention of depressive symptoms and healthy MFA.
