# Supplementary material for: Maternal-fetal attachment and interrelated factors in pregnant women assisted in Primary Health Care
Source: Rev Lat Am Enfermagem. 2024 Oct 25;32:e4404. doi: 10.1590/1518-8345.7104.4404 (PMC11526247; doi:10.1590/1518-8345.7104.4404)
Supplement: Translation

# Translation: Apego materno-fetal y factores interrelacionados en gestantes atendidas en la Atención Primaria de Salud *

**DOI:** 10.1590/1518-8345.7104.4403

**Published:** 2024-10-08

**Authors:** Cássio de Almeida Lima, Maria Fernanda Santos Figueiredo Brito, Lucineia de Pinho, Sélen Jaqueline Souza Ruas, Romerson Brito Messias, Marise Fagundes Silveira

**Affiliations:** 1 Universidade Estadual de Montes Claros, Departamento de Métodos e Técnicas Educacionais, Montes Claros, MG, Brasil; ^2^ Becario de la Coordenação de Aperfeiçoamento de Pessoal de Nível Superior (CAPES), Brasil.; ^3^ Universidade Estadual de Montes Claros, Departamento de Saúde Mental e Saúde Coletiva, Montes Claros, MG, Brasil; ^4^ Faculdade de Saúde e Humanidades Ibituruna, Departamento de Enfermagem, Montes Claros, MG, Brasil; ^5^ Universidade Estadual de Montes Claros, Departamento de Ciências Exatas, Montes Claros, MG, Brasil; ^6^ Becaria del Conselho Nacional de Desenvolvimento Científico e Tecnológico (CNPq), Brasil

**Keywords:** Mujeres Embarazadas, Relaciones Materno-Fetales, Atención Primaria de Salud, Encuestas Epidemiológicas, Análisis Multivariante, Enfermería en Salud Comunitaria

## Abstract

analizar el apego materno-fetal y los factores interrelacionados en gestantes atendidas en la Atención Primaria de Salud.

encuesta epidemiológica seccional, de base poblacional y analítica. Se investigó una muestra de 937 participantes atendidas por equipos de la Estrategia de Salud de la Familia. Se evaluaron apego materno-fetal (resultado), variables sociodemográficas y clínicas, apoyo social, funcionalidad familiar, síntomas depresivos y estrés percibido. Se adoptó un análisis multivariado mediante la modelización con ecuaciones estructurales.

el apego materno-fetal presentó una media de 92,6 (DE=±15,3). El modelo estructural ajustado evidenció que los siguientes factores ejercieron un efecto directo sobre el resultado: semanas gestacionales (β=0,29; p<0,001), aglomeración del domicilio (β=-0,07; p=0,027), síntomas depresivos (β=-0,11; p=0,003), apoyo social (β=0,08; p<0,001) y funcionalidad familiar (β=0,19; p<0,001). Se identificaron efectos indirectos del apoyo social (β=-0,29; p<0,001) y de la funcionalidad familiar (β=-0,20; p<0,001), mediados por los síntomas depresivos.

se identificó un conjunto de interrelaciones entre apego materno-fetal, semanas gestacionales, aglomeración domiciliaria, síntomas depresivos, apoyo social y funcionalidad familiar. Se sugiere que la Estrategia de Salud de la Familia ofrezca una atención prenatal anclada en la integralidad y humanización, que propicie el bienestar biopsicosocial en el embarazo y un apego materno-fetal saludable.

## Destacados

**(1)** El apego materno-fetal debe ser evaluado en la Atención Primaria de Salud.

**(2)** Los síntomas depresivos presentaron una relación negativa con el apego materno-fetal.

**(3)** El apoyo social y la funcionalidad familiar ejercieron un efecto positivo sobre el apego.

**(4)** Una mayor aglomeración domiciliaria tuvo un efecto negativo sobre el resultado.

**(5)** Se recomienda rastrear a las gestantes con depresión, poco apoyo social y familiar.

## Introducción

 El ciclo gravídico es considerado un período de transición que forma parte del proceso de desarrollo, implica un cambio de identidad y redefinición de roles en la vida de la mujer ^(^ 1 ^-^ 2 ^)^ , además de modificaciones hormonales y emocionales ^(^ 3 ^)^ . Se caracteriza como una fase permeada por adaptaciones a los cambios biopsicosociales, en la que la mujer necesita conciliar demandas y desafíos de la maternidad con funciones familiares, conyugales y profesionales desempeñadas socialmente ^(^ 1 ^-^ 2 ^)^ . 

 La adaptación exitosa de la gestante al proceso de transición a la maternidad está íntimamente vinculada al desarrollo del vínculo afectivo con el feto, es decir, la representación mental del feto y los sentimientos de estar vinculada a él ^(^ 4 ^-^ 5 ^)^ . Es en la fase prenatal en la que se inicia la relación de la madre con su hijo: el apego materno-fetal (AMF), que involucra la intensidad de comportamientos de afiliación e interacción con el niño por nacer. La gestante tiende a establecer una conexión afectiva con el feto y vive expectativas relativas al niño que está por venir, las cuales aumentan a lo largo de la gestación ^(^ 3 ^-^ 5 ^)^ . El AMF involucra expectativas, emociones, pensamientos y comportamientos maternos en relación con el feto, el embarazo y la maternidad, siendo un importante predictor del vínculo madre-bebé en el período posparto ^(^ 2 ^,^ 4 ^-^ 5 ^)^ . El apego puede ser un factor predictivo del estado mental puerperal materno, del autocuidado gestacional, de resultados neonatales, del desarrollo infantil de habilidades conductuales y socioemocionales ^(^ 5 ^)^ . Se trata de un constructo multidimensional ^(^ 2 ^-^ 14 ^)^ , permeado por cuestiones sociodemográficas ^(^ 3 ^,^ 7 ^-^ 8 ^,^ 13 ^-^ 14 ^)^ , obstétricas ^(^ 3 ^,^ 7 ^-^ 9 ^,^ 13 ^-^ 14 ^)^ , culturales ^(^ 2 ^,^ 13 ^-^ 14 ^)^ y psicológicas ^(^ 2 ^-^ 14 ^)^ . 

 La investigación del AMF por el enfermero en la atención prenatal del sistema público de salud puede contribuir al rastreo de gestantes en riesgo de desarrollar un apego deficiente, que necesiten un abordaje multiprofesional teniendo en cuenta la calidad del vínculo madre-feto y las relaciones familiares saludables. Es necesario prestar un cuidado holístico a las gestantes usuarias del Sistema Único de Salud (SUS) y de la Atención Primaria de Salud (APS), que también incluya la evaluación del AMF. El examen de este evento debe considerar aspectos interferentes en ambientes socioculturales específicos ^(^ 13 ^-^ 15 ^)^ , como en comunidades cubiertas por la Estrategia de Salud de la Familia (ESF). En Brasil, estas mujeres viven en un contexto disonante de los escenarios de los estudios previos de la literatura internacional ^(^ 4 ^-^ 5 ^,^ 7 ^-^ 12 ^)^ . 

 También es preciso considerar las directrices nacionales que direccionan la línea de cuidado materno-infantil y el cuidado prenatal en Brasil ^(^ 2023 ^)^ . La Política Nacional de Atención Integral a la Salud de la Mujer (PNAISM) incorpora, en un enfoque de género y empoderamiento de las usuarias del SUS, la integralidad y la promoción de la salud como ejes orientadores, aspirando consolidar los avances en el campo de los derechos sexuales y reproductivos ^(^ 17 ^)^ . La *Rede Cegonha* consiste en una red de cuidados dirigidos a la planificación reproductiva y a la atención humanizada a la mujer en el embarazo, el parto y el posparto. Ella busca el nacimiento seguro, el crecimiento y el desarrollo saludables del niño ^(^ 18 ^)^ . 

 En cuanto a las evidencias sobre el AMF, estas son incipientes en la APS, sobre todo en países latinoamericanos y en Brasil, donde son insuficientes las investigaciones epidemiológicas de base poblacional ^(^ 2 ^,^ 14 ^)^ realizadas con gestantes atendidas en los servicios de la ESF. A partir de amplias encuestas epidemiológicas, es posible sugerir mejoras en la atención prenatal, con el objetivo de promover un AMF saludable ^(^ 15 ^)^ , siendo necesarias investigaciones nacionales que evalúen el AMF y sus determinantes simultáneamente en gestantes usuarias de la APS, donde gran parte de ellas es atendida. Los demás trabajos ^(^ 2 ^-^ 14 ^)^ utilizaron técnicas comunes de estadística inferencial, lo que indica la necesidad de investigaciones con análisis más robustos, como la modelización con ecuaciones estructurales (MEE). Por lo tanto, este estudio tuvo como objetivo analizar el AMF y los factores interrelacionados en gestantes atendidas en la APS. 

## Método

### Diseño del estudio

 Se trata de una encuesta epidemiológica observacional con diseño seccional, de base poblacional y analítico, que utilizó datos de un amplio estudio titulado “Evaluación de las condiciones de salud de las gestantes de Montes Claros - MG: estudio longitudinal (Estudio ALGE)”. Se siguieron las recomendaciones del STROBE – *Strengthening the Reporting of Observational Studies in Epidemiology* ^(^ 19 ^)^ . 

### Escenario

 El escenario del estudio fue la ciudad de Montes Claros, situada en la región norte del estado de Minas Gerais (MG), Brasil. Esta ciudad es considerada polo regional y tiene una población estimada en 417.478 habitantes. Los servicios de la ESF local fueron implantados en la década de 1990 y actualmente están organizados en 15 polos. Estos polos contenían un total de 135 equipos de salud de la familia en la época de la investigación (2018-2020), alcanzando una cobertura del 100% de la población ^(^ 20 ^)^ . 

### Tamaño muestral

La población del Estudio ALGE estuvo constituida por las gestantes registradas en los equipos de la ESF de la zona urbana del municipio, en 2018-2020. El tamaño de la muestra fue establecido con el objetivo de estimar parámetros poblacionales con una prevalencia del 50% (para maximizar el tamaño muestral y debido a que el proyecto original contemplaba diversos resultados). Se consideró un intervalo de confianza del 95% (IC 95%) y un nivel de precisión del 2,0%. Se efectuó una corrección para población finita (N=1.661) y se incluyó un incremento del 20% para compensar las posibles no respuestas y pérdidas. Los cálculos evidenciaron la necesidad de participación de, como mínimo, 1.180 gestantes.

Para la selección de la muestra, se consideraron los polos de la ESF de todas las regiones del municipio, que totalizaban 15 en el período de esta investigación y entre los cuales estaban distribuidos 135 equipos de salud de la familia. El número de gestantes muestreadas en cada polo fue proporcional a su representatividad en relación con la población total de gestantes registradas.

Se incluyeron las gestantes que estaban registradas en un equipo de salud de la familia, en cualquier edad gestacional. No se incluyeron a las mujeres que estaban embarazadas de gemelos (ya que esto podría afectar ciertas variables medidas en el proyecto) y aquellas que presentaban compromiso cognitivo, según diagnóstico médico previo informado por el familiar y/o el equipo de la ESF.

 En el presente trabajo se analizaron solo los datos de las mujeres del segundo y terceros trimestres de gestación. Este recorte ocurrió porque el AMF, de la forma medida por la Escala de Apego Materno-Fetal (EAMF) aplicada, es más evidente a partir del segundo trimestre. A medida que ocurre el crecimiento fetal, la gestante puede sentir los nuevos movimientos del bebé, lo que hace que la experiencia sea más corpórea para ella y permite una interacción más vívida con el feto ^(^ 4 ^,^ 13 ^)^ . En este sentido, para este estudio se consideró solo la población de gestantes que se encontraban en el 2º y 3º trimestre de gestación (N=1.218), por lo que el tamaño mínimo de la muestra se estimó en n=930 gestantes. Se mantuvieron los mismos parámetros adoptados para el cálculo del tamaño muestral del Estudio ALGE. Además, el tamaño muestral definido para la presente investigación cumplió con las premisas del tratamiento estadístico de los datos adoptado en la MEE, en la que se recomienda que la muestra tenga al menos 250-500 observaciones ^(^ 21 ^)^ . 

### Instrumentos y variables

Para la caracterización de las participantes de este estudio, se aplicó un cuestionario estructurado que incluyó variables sociodemográficas: rango de edad (hasta 20 años, 21 a 30, más de 30), estado civil (con pareja, sin pareja), color autodeclarado (pardo, negro, blanco, amarillo), escolaridad (educación primaria, secundaria, superior), renta familiar mensual (menor que R$ 1.000,00, R$ 1.001,00 a 2.000,00, superior a R$ 2.000,00), número de habitantes en la residencia, número de habitaciones en el domicilio; y clínicas: trimestre gestacional (segundo, tercero), planificación del embarazo (sí, no), aborto previo (sí, no), semanas de gestación. La variable sociodemográfica aglomeración del domicilio se calculó mediante la razón “número de habitantes en la residencia/número de habitaciones en la residencia”. Las variables semanas gestacionales y aglomeración del domicilio (numéricas y observadas) se incluyeron en el modelo estructural.

También se emplearon instrumentos validados nacionalmente para investigar los siguientes constructos: AMF, síntomas depresivos, estrés percibido, apoyo social y funcionalidad familiar.

 La versión brasileña de la EAMF ^(^ 22 ^)^ se aplicó para evaluar el resultado principal de esta encuesta, el AMF. El AMF se trató como una variable observada, numérica discreta. La EAMF fue elaborada por la Enfermera Mecca S. Cranley, en 1981, en los Estados Unidos de América (EE.UU) ^(^ 23 ^)^ . Contiene 24 ítems respondidos en una escala Likert de uno a cinco puntos (nunca a casi siempre). La puntuación mínima es 24 y la máxima 120. En el proceso de validación de la EAMF para la población brasileña, se constató la unidimensionalidad del constructo y se recomendó su uso mediante la puntuación total. Así, puntuaciones más elevadas indican niveles más altos de AMF ^(^ 22 ^)^ . Se propone la siguiente clasificación: bajo nivel de apego (24 a 47 puntos), medio (48 a 97) y alto (98 a 120) ^(^ 24 ^)^ . Se registró un coeficiente α de Cronbach de 0,874 en el presente trabajo. 

 La Escala de Tamizaje Poblacional para Depresión del Centro de Estudios Epidemiológicos de los EE.UU ( *Center for Epidemiologic Studies Depression Scale* – CES-D), también validada en Brasil ^(^ 25 ^)^ , se empleó para el tamizaje de síntomas depresivos en la muestra de gestantes evaluadas. La CES-D está compuesta por 20 ítems, de los cuales cuatro son positivos, en los que la entrevistada relata la frecuencia de aparición de los síntomas en la última semana. Cada respuesta puede involucrar cuatro grados crecientes de intensidad en una escala de Likert – nunca o raramente, a veces, con frecuencia y siempre – con puntuaciones correspondientes a 0, 1, 2 y 3. La puntuación de los cuatro ítems positivos se invierte y se suma a la puntuación de los demás, lo que da un resultado final que varía de cero a 60 – cuanto más elevado, mayor la intensidad de los síntomas. Se procedió a la categorización en: síntomas depresivos ausentes/ligeros ( *score* <16), moderados ( *score* ≥16 o ≤21) y síntomas graves ( *score* ≥22) ^(^ 25 ^-^ 26 ^)^ . El instrumento tuvo un resultado satisfactorio del α de Cronbach (0,777). 

 El estrés percibido se investigó mediante la Escala de Estrés Percibido ( *Perceived Stress Scale* /PSS-14), validada en Brasil. El instrumento identifica situaciones en la vida del individuo consideradas como estresantes, estableciendo niveles de intensidad. Las preguntas son de naturaleza general y se aplican a cualquier subgrupo poblacional. Contiene 14 ítems sobre la frecuencia con la que ciertos sentimientos y pensamientos ocurrieron en el último mes, con respuestas que varían de cero (nunca) a cuatro (siempre). La puntuación se obtiene invirtiendo las puntuaciones de los ítems positivos y sumando las respuestas de los 14 ítems, con una puntuación total que varía de cero (sin síntomas de estrés) a 56 (síntomas de estrés extremo). La variable se dicotomizó en ausencia de estrés (resultados menores o iguales a 30) y presencia de estrés (mayores de 30) ^(^ 27 ^)^ . Se identificó un α de Cronbach=0,782 para la PSS-14 en esta investigación, un valor apropiado. 

 La presencia de apoyo social se midió mediante la versión brasileña de la Escala de Apoyo Social, que también fue validada en el país. La escala está compuesta por 19 preguntas que comprenden cinco dimensiones: material, afectiva, emocional, interacción social positiva e información. Para cada ítem, el participante indica con qué frecuencia considera cada tipo de apoyo, mediante una escala tipo Likert: nunca (1), raramente (2), a veces (3), casi siempre (4) y siempre (5). Cuanto más cerca de 100 sea la puntuación final, mejor será el apoyo social percibido. La puntuación general de la escala se calculó por la suma total de los 19 ítems y se consideró como elevado apoyo social el resultado superior a 66, que corresponde al segundo tercil ^(^ 28 ^)^ . El instrumento presentó elevada consistencia interna, con un α de Cronbach de 0,960. 

 Para la evaluación de la funcionalidad familiar, se aplicó un instrumento validado nacionalmente, denominado APGAR Familiar ^(^ 29 ^)^ . Este señala el cumplimiento de parámetros básicos definidos por el acrónimo APGAR: A – Adaptación ( *Adaptation* ); P – Participación ( *Participation* ); G – Crecimiento ( *Growth* ); A – Afecto ( *Affection* ); R – Resolución ( *Resolution* ). El cuestionario presenta cinco preguntas con tres posibilidades de respuestas cada una y puntuaciones que varían de cero a dos puntos – siempre (2), algunas veces (1) y nunca (0). Así, se obtiene una suma de cero a diez puntos, que, cuanto más elevada, indica mejor satisfacción del participante con su familia. Se procedió a la categorización en familia funcional (puntuación de 7-10) y familia disfuncional (<6) ^(^ 30 ^)^ . El instrumento obtuvo una adecuada fiabilidad en esta investigación (α de Cronbach=0,872). 

Las variables AMF, síntomas depresivos, estrés percibido, apoyo social y funcionalidad familiar también se analizaron como observadas y numéricas, mediante sus respectivas puntuaciones.

### Recolección de datos

En cuanto al proceso de recolección de datos, inicialmente se hizo contacto con los gestores de la coordinación de la APS del municipio, para sensibilización y explicación sobre el propósito de la investigación. Tras su anuencia, los equipos de salud de la familia también fueron visitados por los investigadores para aclaraciones sobre el estudio. Los profesionales de estos equipos responsables del prenatal proporcionaron una lista de las gestantes de su área de cobertura con los nombres y direcciones de estas. En posesión de estas listas, un equipo de entrevistadores realizó el contacto inicial con las mujeres, cuando hubo un acercamiento con la invitación y sensibilización sobre el estudio, para que posteriormente se agendara y efectuara la recolección de datos.

Los datos fueron recolectados por un equipo multiprofesional del área de la salud y por estudiantes de iniciación científica (cursos de Educación Física, Enfermería y Medicina) entre octubre de 2018 y febrero de 2020, en las unidades de salud de la ESF o en los domicilios de las participantes, según la disponibilidad de ellas. La recolección de datos ocurrió cara a cara, individualmente con cada gestante, con una duración media de una hora. En cuanto al orden de aplicación de los instrumentos, primero se aplicó un cuestionario estructurado para investigar las características sociodemográficas/económicas y clínicas, y a continuación las escalas referentes al AMF, apoyo social, funcionalidad familiar, estrés percibido y síntomas depresivos.

Previamente a la recolección de datos, hubo una capacitación de los entrevistadores, así como un estudio piloto con 36 gestantes registradas en una unidad de la ESF (que no fueron incluidas en los análisis del estudio), con la finalidad de estandarizar los procedimientos de recolección de datos de la investigación.

### Tratamiento y análisis de los datos

Se adoptó el análisis multivariado mediante la MEE. Primero se elaboró un modelo hipotético, con el fin de evaluar las interrelaciones entre el AMF, considerado como resultado principal, y las demás variables investigadas: semanas gestacionales, aglomeración del domicilio, síntomas de depresión, estrés percibido, apoyo social y funcionalidad familiar.

 Según el modelo hipotetizado, la edad gestacional (semanas de gestación) se colocó en una posición de correlación directa con el resultado principal ^(^ 7 ^-^ 8 ^,^ 13 ^-^ 15 ^)^ . Se teorizó que la aglomeración del domicilio también ejerce efectos directos sobre el AMF, por ser un posible marcador de la condición socioeconómica de la gestante ^(^ 4 ^,^ 6 ^,^ 15 ^)^ . El estrés percibido también presenta relación con el AMF ^(^ 5 ^,^ 7 ^,^ 9 ^,^ 15 ^)^ , al igual que los síntomas depresivos ^(^ 4 ^-^ 5 ^,^ 7 ^,^ 9 ^-^ 11 ^,^ 14 ^)^ . El apoyo social posee efectos directos e indirectos sobre el AMF, en una interrelación mediada por el estrés percibido y los síntomas de depresión ^(^ 4 ^-^ 6 ^,^ 12 ^,^ 15 ^)^ . La misma suposición se hizo para el constructo funcionalidad familiar ^(^ 7 ^,^ 14 ^)^ . 

 La Figura 1 ilustra las relaciones directas e indirectas entre las variables investigadas en el modelo propuesto. Las variables observadas están representadas por rectángulos y las correlaciones son indicadas por flechas (de la variable independiente a la dependiente) ^(^ 31 ^)^ . 

**Figura 1:** - Modelo hipotético para analizar los factores interrelacionados con el apego materno-fetal en gestantes atendidas en la Atención Primaria de Salud. Montes Claros, MG, Brasil, 2018-2020

 En el análisis estadístico de los datos, inicialmente, las variables categóricas se describieron mediante sus distribuciones de frecuencias. Las variables numéricas se describieron mediante la media, el desvío estándar (DE), los valores mínimo y máximo, y los coeficientes de asimetría (sk) y curtosis (ku). Valores de sk> 3 y ku> 10 fueron aceptados como indicadores de violación del supuesto de normalidad ^(^ 31 ^)^ . Los valores perdidos ( *missing* ) fueron imputados por la media. 

 A continuación, se ajustó el modelo multivariado mediante la MEE. Se estimaron efectos directos e indirectos representados por coeficientes estandarizados (CEs), cuyas significancias estadísticas fueron evaluadas por la razón crítica (RC), al nivel del 5%. Los CEs estandarizados se interpretaron de la siguiente manera: con efecto pequeño cuando los valores eran cercanos a 0,10, medio cuando cercanos a 0,30 y grande si superiores a 0,50 ^(^ 32 ^)^ . Los efectos indirectos, intermediados por variables intermedias, se calcularon multiplicando los coeficientes de los caminos indirectos del modelo. 

 Para la evaluación de la adecuación del ajuste global del modelo, se apreciaron los siguientes índices: razón del chi-cuadrado por los grados de libertad (x ^2^ /gl), *confirmatory fit index* (CFI), *goodness of fit index* (GFI), *Tucker-Lewis index* (TLI) y *root mean square error of approximation* (RMSEA). El ajuste del modelo se consideró satisfactorio si x ^2^ /df ≤ 5,0; CFI, GFI y TLI ≥ 0,90; RMSEA < 0,10 ^(^ 33 ^)^ . 

 La organización y los análisis estadísticos de los datos se realizaron en el *software* IBM *Statistical Package for the Social Sciences* (SPSS) *Statistics* , versión 23.0 ^®^ . En un momento anterior, los datos recolectados fueron sometidos a un control de calidad y doble verificación. La modelación estructural se procesó en el *software Analysis of Moment Structures* (IBM SPSS Amos 23.0). 

### Aspectos éticos

 El presente trabajo acató las regulaciones éticas internacionales y nacionales de las investigaciones que involucran a seres humanos. El proyecto fue aprobado por el Comité de Ética en Investigación con Seres Humanos de la *Universidade Estadual de Montes Claros* (CAAE 80957817.5.0000.5146, Informe Fundamentado número 2.483.623/2018 e Informe fundamentado número 3.724.531/2019) y obtuvo la concordancia institucional de la Secretaría Municipal de Salud. Las participantes mayores de edad leyeron y firmaron el Consentimiento Informado. Aquellas que tenían menos de 18 años presentaron el Asentimiento Informado, además del Consentimiento Informado firmado por sus responsables legales. 

## Resultados

 Un total de 1.279 gestantes participó de la línea de base del estudio. En el presente trabajo se incluyeron los datos de 937 entrevistadas, que se encontraban en el segundo y tercer trimestres gestacionales ( Figura 2 ). 

 En cuanto a las características sociodemográficas y clínicas de las participantes de la muestra analizada (n=937), 47,7% estaban en el rango de edad de 21 a 30 años, 77,2% refirieron estado civil con pareja, 46,7% tenían ingresos familiares mensuales de hasta 1.000 reales. De las entrevistadas, 55,0% estaban en el 2º trimestre, 61,0% reportaron un embarazo no planificado, 48,7% eran nulíparas y 18,9% refirieron aborto previo. En lo que respecta a los puntajes del AMF, se observó una media de 92,6 (DE=±15,3) y el nivel de apego medio fue el predominante, verificado en 575 (61,4%) gestantes. Las desviaciones de la normalidad no fueron muy severas para los coeficientes de asimetría y curtosis de las variables del modelo estructural ( Tabla 1 ). 

**Figura 2:** - Diagrama de flujo del proceso de selección de la muestra de gestantes analizadas en el estudio. Montes Claros, MG, Brasil, 2018-2020

**Tabla 1:** - Características de las gestantes atendidas en la Atención Primaria de Salud y medidas descriptivas de las variables del modelo estructural (n = 937). Montes Claros, MG, Brasil, 2018-2020

**Variables**		**n** *	**% [IC 95%** ^†^ **]**	
**Rango etario (a** ñ **os)**				
Hasta 20		203	22,4 [19,2-24,4]	
21 a 30		453	47,6 [45,3-51,6]	
Más de 30		281	30,0 [27,1-33,0]	
**Situación conyugal**				
Con pareja		721	77,2 [74,4-79,8]	
Sin pareja		213	22,8 [20,2-25,6]	
**Color autodeclarado**				
Pardo		641	68,8 [65,7-71,7]	
Negro		147	15,8 [13,6-18,3]	
Blanco		102	10,9 [9,1-13,1]	
Amarillo		42	4,5 [3,4-6,0]	
**Escolaridad**				
Educación primaria		141	15,1 [12,8-17,4]	
Educación secundaria		595	63,5 [60,4-55,6]	
Educación superior		200	21,4 [18,8-24,0]	
**Ingreso familiar mensual (R$** ^‡^ **)**				
Hasta 1.000,00		422	46,7 [43,4-50,0]	
1.000,00 a 2.000,00		280	31,0 [28,0-34,0]	
Más de 2.000,00		202	22,3 [19,6-25,0]	
**Trimestre gestacional**				
2º		515	55,0 [51,8-58,2]	
3º		422	45,0 [41,8-48,2]	
**Planificación del embarazo**				
Sí		360	39,0 [359-42,1]	
No		564	61,0 [57,9-64,1]	
**Aborto previo**				
No		742	81,1 [78,6-83,6]	
Sí		173	18,9 [16,4-21,4]	
**Apego materno-fetal**				
Bajo		10	1,1 [0,4-1,8]	
Medio		575	61,4 [59,3-64,5]	
Alto		352	37,5 [34,4-40,6]	
**Funcionalidad familiar**				
Familia funcional		785	84,0 [81,6-86,4]	
Familia disfuncional		149	16,0 [13,6-18,4]	
**Apoyo social**				
Bajo		166	17,9 [15,4-20,4]	
Elevado		760	82,1 [79,6-84,6]	
**Síntomas depresivos**				
Ausentes/ligeros		560	61,3 [58,1-64,5]	
Moderados		142	15,5 [13,2-17,8]	
Graves		212	23,2 [20,5-25,9]	
**Estrés percibido**				
Ausencia de estrés		768	83,1 [80,7-85,5]	
Presencia de estrés		156	16,9 [14,5-18,3]	
**Variables del modelo estructural**	**Media (DE** ^§^ **)**	**Min-Max** ^||^	**Sk** ^¶^	Ku**
Apego materno-fetal	92,6 (15,3)	29,0-120,0	-0,71	1,03
Funcionalidad familiar	8,5 (2,1)	0,0-10,0	-1,72	2,68
Apoyo social	79,3 (18,3)	0,0-94,7	-1,39	1,52
Síntomas depresivos	15,3 (10,1)	0,0-57,0	1,17	1,22
Estrés percibido	23,5 (8,7)	1,0-56,0	0,15	0,57
Semanas gestacionales	26,3 (7,6)	13,6-45,1	0,11	-0,95
Aglomeración del domicilio	0,7 (0,3)	0,20-3,50	2,22	8,31

* n varía debido a la ausencia de información ( *missing* )

^†^ IC 95% = Intervalo de confianza del 95%

^‡^ R$ = Real brasileño (salario mínimo: R$ 954,00)

^§^ DE = Desviación-estándar

^||^ Min-Max = Mínimo y máximo

^¶^ sk = Asimetría

**sk = Asimetría:** ku = Curtosis

 La Figura 3 presenta las interrelaciones entre los factores investigados y los coeficientes estructurales estandarizados. El modelo estructural ajustado obtuvo indicadores de calidad de ajuste adecuados, con los siguientes valores: x ^2^ /gl=2,29, GFI=0,994, CFI=0,989, TLI=0,975, RMSEA=0,037 (IC 90%=0,016; 0,059, p-valor=0,823). De acuerdo con el modelo, las semanas de gestación ejercieron un efecto directo positivo sobre el resultado AMF (β=0,29; p<0,001), mientras que la aglomeración del domicilio mostró un efecto directo negativo (β=-0,07; p=0,027). Los síntomas depresivos tuvieron un efecto directo negativo sobre el resultado (β= 0,11; p=0,003). El apoyo social presentó una correlación positiva y directa con el apego (β=0,08; p<0,001), además de un efecto indirecto negativo mediado por la variable síntomas depresivos (β=-0,29; p<0,001). En cuanto a la funcionalidad familiar, también se observó un efecto directo positivo sobre el AMF (β=0,19; p<0,001), así como un efecto negativo en interrelación con los síntomas depresivos (β=-0,20; p<0,001). 

 Las magnitudes de los efectos directo, indirecto y total están descritas en la Tabla 2 . En el análisis de mediación, solo el estrés percibido no obtuvo evidencia estadística de relación con el evento analizado. 

**Figura 3:** - Modelo estructural ajustado de los factores interrelacionados con el apego materno-fetal en gestantes atendidas en la Atención Primaria de Salud (n = 937). Montes Claros, MG, Brasil, 2018-2020

**Tabla 2:** - Magnitudes de los efectos directo, indirecto y total estimados de los factores interrelacionados con el apego materno-fetal en gestantes atendidas en la Atención Primaria de Salud (n = 937). Montes Claros, MG, Brasil, 2018-2020

**Variable independiente**	**Efecto**	**Variable dependiente**	**Coeficiente estandarizado**	**Total**	Valor de p*
	**Directo/Indirecto**		**Directo/Indirecto**		
Semanas gestacionales	Directo	Apego materno-fetal	0,29	0,29	<0,001
Aglomeración domiciliar	Directo	Apego materno-fetal	-0,07	-0,07	0,027
Síntomas depresivos	Directo	Apego materno-fetal	-0,11	-0,11	0,003
Estrés percibido	Directo	Apego materno-fetal	-0,02	-0,02	0,539
Apoyo social	Directo	Apego materno-fetal	0,08		
	Indirecto vía síntomas depresivos		-0,29x(-0,11)=0,0319	0,12	<0,001
	Indirecto vía estrés percibido		-0,29x(-0,02)=0,0058		
	Directo	Síntomas depresivos	-0,29	-0,29	<0,001
	Directo	Estrés percibido	-0,29	-0,29	<0,001
Funcionalidad familiar	Directo	Apego materno-fetal	0,19		
	Indirecto vía síntomas depresivos		-0,20x(-0,11)=0,022	0,22	<0,001
	Indirecto vía estrés percibido		-0,17x(-0,02)=0,0034		
	Directo	Síntomas depresivos	-0,20	-0,20	<0,001
	Directo	Estrés percibido	-0,17	-0,17	<0,001

* Nivel de significancia estadística (p<0,05)

## Discusión

El presente estudio evidenció las interrelaciones entre AMF, edad gestacional, aglomeración domiciliaria, síntomas depresivos, apoyo social y funcionalidad familiar en gestantes brasileñas atendidas en la APS. Por intermedio de la MEE, fue posible comprender un sistema compuesto por múltiples factores y sus interrelaciones.

 En el modelo estructural ajustado, se observó que las semanas de gestación tienen una relación directa con el evento investigado, mostrando ser un resultado positivo. Un resultado similar se identificó en investigaciones con gestantes italianas ^(^ 8 ^)^ y brasileñas en la región sur del país ^(^ 13 ^-^ 14 ^)^ . Se trata de un hallazgo plausible, ya que los comportamientos de AMF aumentan con el avance de la edad gestacional a medida que la mujer siente los movimientos fetales, lo que hace que la experiencia sea más corporal para ella y acentúa la interacción madre-bebé. A partir del cuarto mes de gestación, con los movimientos del feto, la madre comienza a expresar de manera más intensa representaciones, expectativas, preocupaciones y afectos hacia el hijo por nacer ^(^ 8 ^,^ 13 ^-^ 14 ^)^ . 

 Por otro lado, la aglomeración del domicilio presentó una relación negativa con el AMF en las gestantes entrevistadas: hubo un comportamiento de disminución de los puntajes de apego a medida que aumentaba la aglomeración de personas en la residencia. Este factor puede considerarse un marcador del *estatus* socioeconómico de la madre y de su familia, lo cual compromete la calidad del vínculo afectivo madre-feto. Ambientes domésticos positivos, estables y estimulantes permiten que la relación madre-feto se establezca de forma más saludable. Cuando esta situación no está presente, puede influir negativamente en la calidad de la interacción materna, ya que constituye una fuente de estrés familiar ^(^ 6 ^)^ . Las bajas condiciones socioeconómicas y los contextos de vida desfavorables pueden perjudicar la adaptación materna y familiar al embarazo, ya que la gestación requiere una reestructuración en varias dimensiones ^(^ 3 ^)^ . 

 Otro aspecto negativo en esta encuesta epidemiológica fue la presencia de síntomas depresivos, que se correlacionaron negativamente con el AMF. Este hallazgo ha sido evidenciado en la literatura internacional, en países como Dinamarca ^(^ 4 ^,^ 34 ^)^ , China ^(^ 5 ^)^ , Irán ^(^ 11 ^)^ , Turquía ^(^ 9 ^)^ y Portugal ^(^ 10 ^)^ ; así como en una revisión sistemática ^(^ 7 ^)^ . En Brasil, también se observó en una investigación de base domiciliaria en la región Sur ^(^ 14 ^)^ . El embarazo puede no constituir un factor de protección para la salud mental materna, por el contrario, puede situar a la mujer en una posición de vulnerabilidad al sufrimiento psíquico ^(^ 1 ^)^ . Los múltiples cambios vividos en el período gravídico exigen adaptaciones y pueden favorecer el desarrollo de psicopatologías ^(^ 3 ^)^ , como la depresión, que es común en esta fase ^(^ 7 ^)^ . Esta condición puede interferir en el vínculo afectivo de la gestante con el feto en el prenatal ^(^ 3 ^,^ 14 ^,^ 34 ^)^ . Se teoriza que la depresión afecta la capacidad empática y la disponibilidad de afecto materno, por lo que la gestante puede considerar al feto como una fuente de irritación o culpa, resultando en un estado de distanciamiento ^(^ 14 ^,^ 34 ^)^ . Sin embargo, no es posible asegurar una relación causal directa entre la depresión y el AMF, dada la naturaleza subjetiva y singular de estas condiciones, lo que requiere cautela en la interpretación del resultado observado. Las investigaciones cualitativas y longitudinales pueden contribuir a profundizar en la comprensión de este hallazgo. 

 Las gestantes con sintomatología depresiva tienden a experimentar la maternidad como sombría y llena de sentimientos de culpa, tristeza, fatiga, baja autoestima y desesperanza. Puede suceder que la madre carezca de confianza en realizar las actividades cotidianas y en cuidar del futuro bebé, según lo deseado, además de sentir miedo, tensión, pensamientos intrusivos de amenaza a la vida o al bienestar del hijo. Se configura una situación generadora de sufrimiento y angustia para la mujer, que incluso puede ocultar su estado mental por temor a ser estigmatizada. Existe un impacto negativo en la salud materno-infantil, ya que los síntomas depresivos pueden continuar en el puerperio ^(^ 5 ^,^ 15 ^,^ 35 ^)^ . Los hijos de madres deprimidas pueden presentar dificultades en el desarrollo afectivo, social y cognitivo ^(^ 1 ^)^ . Por lo tanto, en el ámbito de la atención de enfermería en la ESF, se recomienda la identificación precoz de mujeres en vulnerabilidad psicológica ^(^ 15 ^)^ , así como la atención a la salud mental de las gestantes en la atención prenatal, con el fin de prevenir el cuadro depresivo ^(^ 6 ^)^ . También es necesario el cuidado humanizado, como lo recomienda el Ministerio de Salud de Brasil en sus directrices oficiales ^(^ 17 ^-^ 18 ^,^ 36 ^-^ 37 ^)^ . 

 La asistencia profesional ampliada, resolutiva y humanizada es importante, especialmente al considerar que, en esta investigación, hubo evidencia estadística de una relación positiva y directa entre el apoyo social y el apego: un mayor nivel de apoyo social repercutió en una mayor intensidad de AMF. Se observó un efecto indirecto negativo mediado por la variable síntomas depresivos en la interrelación entre apoyo social y el resultado principal, es decir, cuanto mayor es el apoyo social de la gestante, menor es la intensidad de los síntomas de depresión, los cuales impactaron negativamente en el vínculo afectivo madre-feto. La conexión entre apoyo social y AMF también fue registrada en estudios realizados con gestantes danesas ^(^ 4 ^)^ , iraníes ^(^ 11 ^)^ y turcas ^(^ 9 ^)^ . El elevado apoyo social puede contribuir al bienestar del binomio madre-feto, ya que las gestantes que tienen una red de apoyo y cuidados presumiblemente son más propensas al autocuidado y al vínculo afectivo con el feto ^(^ 14 ^)^ . Este constructo es importante para el mantenimiento de la salud mental, el afrontamiento de situaciones estresantes y la adecuación de los comportamientos maternos requeridos en la fase gravídico-puerperal. Un buen apoyo social puede ser un aspecto que protege contra la aparición de síntomas depresivos y promueve una maternidad saludable. El papel de este apoyo es fundamental para amortiguar los factores estresantes que ocurren en la vida cotidiana, especialmente en el caso del embarazo, cuando ocurren diversas modificaciones psicosociales y fisiológicas ^(^ 6 ^,^ 38 ^)^ . 

 En cuanto a la funcionalidad familiar, esta investigación mostró un resultado beneficioso similar al del apoyo social. Se observó un efecto directo positivo sobre el AMF, así como un efecto mediado por los síntomas depresivos. Se infirió que una adecuada funcionalidad familiar implica un mejor AMF y una menor intensidad de síntomas depresivos, lo que evidencia la repercusión favorable de una red familiar saludable sobre el evento en análisis. Durante el período gestacional, la mujer necesita el apoyo de la familia para poder adaptarse a las nuevas condiciones derivadas de la transición a la maternidad ^(^ 11 ^)^ . Esta base, especialmente en momentos de vulnerabilidad en el ciclo vital, ha demostrado ser un factor de protección para la salud mental materna ^(^ 1 ^,^ 14 ^)^ . La relación familiar satisfactoria revela una mayor percepción de apoyo práctico y emocional, fomenta sentimientos de pertenencia y potencia el desempeño de la función materna ^(^ 1 ^)^ . Esto contribuye directamente a un contexto de vida más propicio para el establecimiento del AMF. En el escenario comunitario, se recomienda a los enfermeros y a los equipos de la ESF responsables de la atención prenatal una orientación centrada en la promoción del bienestar psicosocial y las relaciones familiares saludables de las gestantes ^(^ 15 ^)^ , según lo estipulado en la Política Nacional de Atención Básica (PNAB) ^(^ 36 ^)^ y en las recomendaciones para el prenatal de bajo riesgo ^(^ 37 ^)^ . 

 Ante los hallazgos identificados en este estudio, se informa que existe un conjunto de evidencias internacionales ^(^ 4 ^-^ 5 ^,^ 9 ^,^ 11 ^,^ 34 ^)^ y brasileñas ^(^ 2 ^-^ 3 ^,^ 13 ^-^ 14 ^,^ 35 ^,^ 38 ^)^ que pueden guiar la práctica del enfermero de salud de la familia. Estas evidencias orientan la detección de gestantes usuarias de los servicios de la APS en situación de vulnerabilidad, con síntomas de depresión, dificultad de adaptación al embarazo, poco apoyo social y familiar, que requieren cuidados dirigidos a la promoción de la salud mental. Es necesario proporcionar un mayor apoyo psicosocial y emocional a las gestantes mediante un enfoque más holístico por parte de los equipos de la ESF, lo que puede influir positivamente en el AMF ^(^ 5 ^,^ 9 ^,^ 15 ^,^ 35 ^,^ 39 ^)^ . Además de los aspectos físicos, la dimensión subjetiva que permea el período gestacional, como el AMF, debe ser considerada por el enfermero y otros profesionales de la salud de la familia, ya que está asociada con mejores resultados materno-infantiles en el período posparto. Es necesario que estos profesionales evalúen el apego y lo estimulen precozmente. Los grupos de educación en salud con las gestantes en el prenatal ofrecido en las unidades de la ESF pueden ser beneficiosos, ya que permiten a las participantes compartir miedos, angustias y expectativas, brindando una sensación de apoyo ^(^ 2 ^,^ 39 ^)^ . 

 Teniendo en cuenta la promoción del cuidado humanizado, resolutivo e integral, así como la calidad de vida del binomio materno-fetal, se recomienda a los enfermeros de los equipos de la ESF que la atención prenatal se base en las directrices nacionales del Ministerio de Salud que orientan la línea de cuidado materno-infantil. Se pueden destacar la PNAISM ^(^ 17 ^)^ , la *Rede Cegonha* ^(^ 18 ^)^ , la PNAB ^(^ 36 ^)^ y las orientaciones presentes en el documento *Cadernos de Atenção Básica* sobre la práctica profesional en la atención prenatal de bajo riesgo ^(^ 37 ^)^ . En la cotidianidad de la atención en salud, otra sugerencia es la aplicación de la EAMF en las consultas y en los grupos de prenatal, con el fin de identificar a las gestantes que tienen dificultades para establecer un vínculo afectivo con el feto. En el ámbito de la ESF y del SUS, es posible detectar precozmente a las gestantes y familias que necesiten una intervención profesional, con objetivo de promover la calidad del vínculo madre-feto ^(^ 15 ^)^ . 

Este trabajo tiene ciertas limitaciones. La información fue autoinformada por las participantes, por lo que está sujeta al sesgo de aceptabilidad social y al sesgo de memoria. Aunque el exceso de variables en el modelo pueda afectar la calidad del ajuste, no se analizaron otras variables, principalmente relativas al perfil socioeconómico, religioso y al estilo de vida. Se sugiere realizar otras investigaciones con un diseño longitudinal prospectivo, para examinar la relación causal entre los factores verificados y el AMF, con la adición de las variables mencionadas. Otra recomendación es la inclusión de gestantes de áreas rurales en estudios futuros.

A pesar de estas limitaciones, vale la pena destacar aspectos positivos de la presente investigación. Se trató de una amplia encuesta epidemiológica de base poblacional, con una muestra significativa y una cobertura de toda la zona urbana cubierta por los equipos de la ESF. Se aplicaron instrumentos validados en Brasil, lo que confiere mayor fiabilidad a los resultados. El análisis con MEE proporcionó mayor solidez y consistencia a la investigación. Los hallazgos obtenidos pueden contribuir a agregar evidencias epidemiológicas sobre el tema en el contexto de la APS, especialmente al reconocer la novedad nacional de esta encuesta, al involucrar a gestantes atendidas por equipos de salud de la familia.

## Conclusión

El presente estudio evidenció una red de interrelaciones entre factores predictores del AMF en gestantes atendidas por equipos de la ESF. El modelo estructural ajustado reveló que una mayor edad gestacional, un mayor apoyo social y una mejor funcionalidad familiar tenían una relación positiva con el AMF. La mayor aglomeración del domicilio y los síntomas depresivos influyeron negativamente en este constructo. Se señaló un papel protector del adecuado apoyo social y familiar para la salud mental de las gestantes. Estos resultados son importantes para los enfermeros de la APS que realizan la atención prenatal, ya que indican la necesidad de una atención anclada en la integralidad y humanización, que propicie el bienestar biopsicosocial, la salud mental, la prevención de síntomas depresivos y un AMF saludable.

# Translation: Apego materno-fetal e fatores inter-relacionados em gestantes assistidas na Atenção Primária à Saúde *

**DOI:** 10.1590/1518-8345.7104.4405

**Published:** 2024-10-08

**Authors:** Cássio de Almeida Lima, Maria Fernanda Santos Figueiredo Brito, Lucineia de Pinho, Sélen Jaqueline Souza Ruas, Romerson Brito Messias, Marise Fagundes Silveira

**Affiliations:** 1 Universidade Estadual de Montes Claros, Departamento de Métodos e Técnicas Educacionais, Montes Claros, MG, Brasil; ^2^ Bolsista da Coordenação de Aperfeiçoamento de Pessoal de Nível Superior (CAPES), Brasil.; ^3^ Universidade Estadual de Montes Claros, Departamento de Saúde Mental e Saúde Coletiva, Montes Claros, MG, Brasil; ^4^ Faculdade de Saúde e Humanidades Ibituruna, Departamento de Enfermagem, Montes Claros, MG, Brasil; ^5^ Universidade Estadual de Montes Claros, Departamento de Ciências Exatas, Montes Claros, MG, Brasil; ^6^ Bolsista do Conselho Nacional de Desenvolvimento Científico e Tecnológico (CNPq), Brasil.

**Keywords:** Gestantes, Relações Materno-Fetais, Atenção Primária à Saúde, Inquéritos Epidemiológicos, Análise Multivariada, Enfermagem em Saúde Comunitária

## Abstract

analisar o apego materno-fetal e os fatores inter-relacionados em gestantes assistidas na Atenção Primária à Saúde.

inquérito epidemiológico seccional, de base populacional e analítico. Foi investigada a amostra de 937 participantes, assistidas por equipes da Estratégia Saúde da Família. Avaliaram-se apego materno-fetal (desfecho), variáveis sociodemográficas e clínicas, apoio social, funcionalidade familiar, sintomas depressivos e estresse percebido. Foi adotada análise multivariada por meio da modelagem com equações estruturais.

o apego materno-fetal apresentou média de 92,6 (DP=±15,3). O modelo estrutural ajustado evidenciou que os seguintes fatores exerceram efeito direto sobre o desfecho: semanas gestacionais (β=0,29; p<0,001), aglomeração do domicílio (β=-0,07; p=0,027), sintomas depressivos (β=-0,11; p=0,003), apoio social (β=0,08; p<0,001) e funcionalidade familiar (β=0,19; p<0,001). Foram identificados efeitos indiretos do apoio social (β=-0,29; p<0,001) e da funcionalidade familiar (β=-0,20; p<0,001), mediados pelos sintomas depressivos.

identificou-se um conjunto de inter-relações entre apego materno-fetal, semanas gestacionais, aglomeração domiciliar, sintomas depressivos, apoio social e funcionalidade familiar. Sugere-se que a Estratégia Saúde da Família oferte uma atenção pré-natal ancorada na integralidade e humanização, que propicie o bem-estar biopsicossocial na gravidez e o apego materno-fetal saudável.

## Destaques

**(1)** O apego materno-fetal deve ser avaliado na Atenção Primária à Saúde.

**(2)** Os sintomas depressivos apresentaram relação negativa com o apego materno-fetal.

**(3)** Apoio social e funcionalidade familiar exerceram efeito positivo sobre o apego.

**(4)** Maior aglomeração domiciliar teve efeito negativo sobre o desfecho.

**(5)** Recomenda-se rastrear gestantes com depressão, com pouco apoio social e familiar.

## Introdução

 O ciclo gravídico é considerado um período de transição que faz parte do processo de desenvolvimento, envolve mudança de identidade e redefinição de papéis na vida da mulher ^(^ 1 ^-^ 2 ^)^ , além de modificações hormonais e emocionais ^(^ 3 ^)^ . Caracteriza-se como uma fase permeada por adaptações às mudanças biopsicossociais, em que a mulher precisa conciliar demandas e desafios da maternidade com funções familiares, conjugais e profissionais desempenhadas socialmente ^(^ 1 ^-^ 2 ^)^ . 

 A adaptação bem-sucedida da gestante ao processo de transição para a maternidade está intimamente ligada ao desenvolvimento do vínculo afetivo com o feto, ou seja, a representação mental do feto e os sentimentos de estar ligada a ele ^(^ 4 ^-^ 5 ^)^ . É na fase pré-natal em que se inicia a relação da mãe com seu filho: o apego materno-fetal (AMF), que envolve a intensidade de comportamentos de afiliação e interação com a criança por nascer. A gestante tende a estabelecer uma conexão afetiva com o feto e vivencia expectativas relativas à criança que está por vir, as quais aumentam ao longo da gestação ^(^ 3 ^-^ 5 ^)^ . O AMF envolve expectativas, emoções, pensamentos e comportamentos maternos em relação ao feto, à gravidez e à maternidade, sendo um importante preditor do vínculo mãe-bebê no período pós-parto ^(^ 2 ^,^ 4 ^-^ 5 ^)^ . Logo, o apego pode ser um fator preditivo do estado mental puerperal materno, do autocuidado gestacional, de resultados neonatais, do desenvolvimento infantil de habilidades comportamentais e socioemocionais ^(^ 5 ^)^ . Trata-se de um construto multidimensional ^(^ 2 ^-^ 14 ^)^ , permeado por questões sociodemográficas ^(^ 3 ^,^ 7 ^-^ 8 ^,^ 13 ^-^ 14 ^)^ , obstétricas ^(^ 3 ^,^ 7 ^-^ 9 ^,^ 13 ^-^ 14 ^)^ , culturais ^(^ 2 ^,^ 13 ^-^ 14 ^)^ e psicológicas ^(^ 2 ^-^ 14 ^)^ . 

 Consequentemente, a investigação do AMF pelo enfermeiro na atenção pré-natal do sistema público de saúde pode contribuir para o rastreio de gestantes em risco de desenvolver um apego deficiente, que necessitem de abordagem multiprofissional, tendo em vista a qualidade do vínculo mãe-feto e as relações familiares saudáveis. É necessário prestar um cuidado holístico às gestantes usuárias do Sistema Único de Saúde (SUS) e da Atenção Primária à Saúde (APS), que também englobe a avaliação do AMF. O exame desse evento deve considerar aspectos interferentes em ambientes socioculturais específicos ^(^ 13 ^-^ 15 ^)^ , como em comunidades cobertas pela Estratégia Saúde da Família (ESF). No Brasil, essas mulheres vivem em um contexto dissonante dos cenários dos estudos prévios da literatura internacional ^(^ 4 ^-^ 5 ^,^ 7 ^-^ 12 ^)^ . 

 Também é preciso considerar as diretrizes nacionais que direcionam a linha de cuidado materno-infantil e o cuidado pré-natal no Brasil ^(^ 2023 ^)^ . A Política Nacional de Atenção Integral à Saúde da Mulher (PNAISM) incorpora, em um viés de gênero e de empoderamento das usuárias do SUS, a integralidade e a promoção da saúde como eixos norteadores, aspirando consolidar os avanços no campo dos direitos sexuais e reprodutivos ^(^ 17 ^)^ . A Rede Cegonha consiste em uma rede de cuidados direcionados ao planejamento reprodutivo e à atenção humanizada à mulher na gravidez, no parto e no puerpério. Ela visa o nascimento seguro, o crescimento e o desenvolvimento saudáveis da criança ^(^ 18 ^)^ . 

 No tocante às evidências sobre o AMF, estas são incipientes na APS, sobretudo em países latino-americanos e no Brasil, onde são insuficientes as pesquisas epidemiológicas de base populacional ^(^ 2 ^,^ 14 ^)^ realizadas com gestantes assistidas nos serviços da ESF. A partir de amplos inquéritos epidemiológicos, é possível sugerir melhorias na atenção pré-natal, com vistas à promoção do AMF saudável ^(^ 15 ^)^ , tornando-se necessárias investigações nacionais que avaliem o AMF e seus determinantes simultaneamente em gestantes usuárias da APS, pela qual grande parte delas é atendida. Os demais trabalhos ^(^ 2 ^-^ 14 ^)^ utilizaram técnicas comuns de estatística inferencial, o que indica a necessidade de pesquisas com análises mais robustas, como a modelagem com equações estruturais (MEE). Portanto, este estudo teve como objetivo analisar o AMF e os fatores inter-relacionados em gestantes assistidas na APS. 

## Método

### Delineamento do estudo

 Trata-se de um inquérito epidemiológico observacional com desenho seccional, de base populacional e analítico, que utilizou dados de um amplo estudo intitulado “Avaliação das condições de saúde das gestantes de Montes Claros - MG: estudo longitudinal (Estudo ALGE)”. As recomendações do STROBE – *Strengthening the Reporting of Observational Studies in Epidemiology* ^(^ 19 ^)^ foram obedecidas. 

### Cenário

 O cenário do estudo foi a cidade de Montes Claros, situada na região norte do estado de Minas Gerais (MG), Brasil. Essa cidade é considerada polo regional e possui população estimada em 417.478 habitantes. Os serviços da ESF local foram implantados na década de 1990 e atualmente estão organizados em 15 polos. Esses polos continham o total de 135 equipes de saúde da família à época da investigação (2018-2020), perfazendo uma cobertura de 100% da população ^(^ 20 ^)^ . 

### Tamanho amostral

A população do Estudo ALGE foi constituída pelas gestantes cadastradas nas equipes da ESF, da zona urbana do município, em 2018-2020. O tamanho da amostra foi estabelecido visando a estimar parâmetros populacionais com prevalência de 50% (para maximizar o tamanho amostral e devido ao projeto original contemplar diversos desfechos). Considerou-se intervalo de 95% de confiança (IC 95%) e nível de precisão de 2,0%. Além do que, foi efetuada correção para população finita (N=1.661) e se incluiu um acréscimo de 20% para compensar as possíveis não respostas e perdas. Os cálculos evidenciaram a necessidade de participação de, no mínimo, 1.180 gestantes.

Para a seleção da amostra, foram considerados os polos da ESF de todas as regiões do município, que totalizavam 15 no período desta pesquisa, e entre os quais estavam distribuídas 135 equipes de saúde da família. O número de gestantes amostradas em cada polo foi proporcional à sua representatividade em relação à população total de gestantes cadastradas.

Foram incluídas as gestantes que estavam cadastradas em equipe de saúde da família em qualquer idade gestacional. No entanto, não foram inseridas as mulheres que estavam grávidas de gemelares (pois isso poderia afetar certas variáveis aferidas no projeto) e as que apresentavam comprometimento cognitivo, conforme diagnóstico médico prévio informado pelo familiar e/ou pela equipe da ESF.

 No presente trabalho foram analisados somente os dados das mulheres do segundo e terceiro trimestres gravídicos. Esse recorte ocorreu porque o AMF, da forma mensurada pela Escala de Apego Materno-Fetal (EAMF) aplicada, é mais evidente a partir do segundo trimestre. À medida que ocorre o crescimento fetal, a gestante pode sentir os novos movimentos do bebê, o que torna a experiência mais corpórea para ela e permite uma interação mais vívida com o feto ^(^ 4 ^,^ 13 ^)^ . Nesse sentido, para este estudo foi considerada somente a população de gestantes que se encontravam nos 2º e 3º trimestre gestacionais (N=1.218), sendo assim, o tamanho mínimo da amostra foi estimado em n=930 gestantes. Mantiveram-se os mesmos parâmetros adotados para o cálculo do tamanho amostral do Estudo ALGE. Ademais, o tamanho amostral definido para a presente investigação atendeu as premissas do tratamento estatístico dos dados adotado na MEE, na qual se recomenda que a amostra tenha pelo menos 250-500 observações ^(^ 21 ^)^ . 

### Instrumentos e variáveis

Para a caracterização das participantes deste estudo, aplicou-se um questionário estruturado que contemplou variáveis sociodemográficas – faixa etária (até 20 anos; 21 a 30; acima de 30), estado conjugal (com companheiro; sem companheiro), cor autodeclarada (parda; negra; branca; amarela), escolaridade (ensino fundamental; médio; superior), renda familiar mensal (menor que R$ 1.000,00; R$ 1.001,00 a 2.000,00; superior a R$ 2.000,00), número de moradores na residência e número de cômodos no domicílio; e clínicas – trimestre gestacional (segundo; terceiro), planejamento da gestação (sim; não), aborto prévio (sim; não) e semanas de gestação. A variável sociodemográfica aglomeração do domicílio foi calculada pela razão “número de moradores na residência/número de cômodos na residência”. Enquanto que as variáveis semanas gestacionais e aglomeração do domicílio (numéricas e observadas) foram incluídas no modelo estrutural.

Também foram empregados instrumentos validados nacionalmente para investigar os seguintes construtos: AMF, sintomas depressivos, estresse percebido, apoio social e funcionalidade familiar.

 A versão brasileira da EAMF ^(^ 22 ^)^ foi aplicada para avaliar o desfecho principal deste inquérito, o AMF. O AMF foi tratado como variável observada, numérica discreta. A EAMF foi elaborada pela Enfermeira Mecca S. Cranley, em 1981, nos Estados Unidos da América (EUA) ^(^ 23 ^)^ . Ela contém 24 itens respondidos em escala Likert de um a cinco pontos (nunca a quase sempre). Posto isto, a pontuação mínima é 24 e a máxima 120. No processo de validação da EAMF para a população brasileira, constatou-se a unidimensionalidade do construto e foi recomendado seu uso mediante a pontuação total. Assim, pontuações mais elevadas indicam níveis mais altos de AMF ^(^ 22 ^)^ . Logo, é proposta a seguinte classificação: baixo nível de apego (24 a 47 pontos), médio (48 a 97) e alto (98 a 120) ^(^ 24 ^)^ . Registrou-se o coeficiente α de Cronbach de 0,874 no presente trabalho. 

 Outrossim, a Escala de Rastreamento Populacional para Depressão do Centro de Estudos Epidemiológicos dos EUA ( *Center for Epidemiologic Studies Depression Scale* – CES-D), também validada no Brasil ^(^ 25 ^)^ , foi empregada para o rastreio de sintomas depressivos na amostra de gestantes avaliadas. A CES-D é composta por 20 itens, dos quais quatro são positivos, em que a entrevistada relata a frequência da ocorrência dos sintomas na última semana. Cada resposta pode envolver quatro graus crescentes de intensidade em uma escala de Likert *–* nunca ou raramente, às vezes, com frequência e sempre – com pontuações correspondentes a zero, um, dois e três. O escore dos quatro itens positivos é invertido e somado ao escore dos demais, o que perfaz um resultado final que varia de zero a 60 – quanto mais elevado, maior a intensidade de sintomas. Por conseguinte, procedeu-se à categorização em: sintomas depressivos ausentes/leves (escore<16), moderados (escore ≥16 ou ≤21) e sintomas graves (escore ≥22) ^(^ 25 ^-^ 26 ^)^ . O instrumento teve resultado satisfatório do α de Cronbach (0,777). 

 O estresse percebido foi averiguado por intermédio da Escala de Estresse Percebido ( *Perceveid Stress Scale* /PSS-14), validada no Brasil. O instrumento identifica situações na vida do indivíduo julgadas como estressantes, estabelecendo níveis de intensidade. As questões são de natureza geral e se aplicam a qualquer subgrupo populacional e, ainda, contém 14 itens sobre a frequência em que determinados sentimentos e pensamentos ocorreram no último mês, com respostas que variam de zero (nunca) a quatro (sempre). O escore é obtido revertendo-se os escores dos itens positivos e somando-se as respostas dos 14 itens, com escore total que varia de zero (sem sintomas de estresse) a 56 (sintomas de estresse extremo). Consequentemente, a variável foi dicotomizada em ausência de estresse (resultados menores ou iguais a 30) e presença de estresse (acima de 30) ^(^ 27 ^)^ . Identificou-se α de Cronbach=0,782 para a PSS-14 nesta pesquisa, um valor apropriado. 

 Por outro lado, a presença de apoio social foi medida mediante a versão brasileira da Escala de Apoio Social, que também passou pela validação no país. A escala é composta por 19 questões que compreendem cinco dimensões: material, afetiva, emocional, interação social positiva e informação. Para cada item, o participante indica com que frequência considera cada tipo de apoio, por meio de uma escala tipo Likert: nunca (1), raramente (2), às vezes (3), quase sempre (4) e sempre (5). Quanto mais próximo de 100 for o escore final, melhor o apoio social percebido. À vista disso, o escore geral da escala foi calculado pela soma total dos 19 itens e se considerou como elevado apoio social o resultado superior a 66, que corresponde ao segundo tercil ^(^ 28 ^)^ . O instrumento apresentou elevada consistência interna, com α de Cronbach de 0,960. 

 Para a avaliação da funcionalidade familiar, aplicou-se um instrumento validado nacionalmente, nomeado APGAR Familiar ^(^ 29 ^)^ . Ele sinaliza o cumprimento de parâmetros básicos definidos pelo acrônimo APGAR: A – Adaptação ( *Adaptation* ); P – Participação ( *Participation* ); G – Crescimento ( *Growth* ); A – Afeição ( *Affection* ); R – Resolução ( *Resolution* ). O questionário apresenta cinco perguntas, com três possibilidades de respostas cada uma, e pontuação que varia de zero a dois pontos – sempre (2), algumas vezes (1) e nunca (0). Desse modo, é dado o somatório de zero a dez pontos, que, quanto mais elevado, aponta melhor satisfação do participante com sua família. Logo, procedeu-se à categorização em família funcional (pontuação de 7-10) e família disfuncional (<6) ^(^ 30 ^)^ . O instrumento obteve adequada confiabilidade nesta investigação (α de Cronbach=0,872). 

Mediante o exposto, as variáveis AMF, sintomas depressivos, estresse percebido, apoio social e funcionalidade familiar também foram analisadas como observadas e numéricas, por meio de seus respectivos escores.

### Coleta de dados

Quanto ao processo de coleta de dados, inicialmente fez-se contato com os gestores da coordenação da APS do município, para sensibilização e explicação sobre o propósito da pesquisa. Após a sua anuência, as equipes de saúde da família também foram visitadas pelos pesquisadores para esclarecimentos sobre o estudo. Os profissionais dessas equipes responsáveis pelo pré-natal forneceram uma lista das gestantes de sua área de abrangência, contendo os nomes e endereços destas. De posse dessas listas, uma equipe de entrevistadores realizou o contato inicial com as mulheres, foi quando houve uma abordagem com o convite e a sensibilização sobre o estudo, para que em seguida fosse agendada e efetuada a coleta de dados.

Com efeito, os dados foram coletados por uma equipe multiprofissional da área da saúde e por acadêmicos de iniciação científica (cursos de Educação Física, Enfermagem e Medicina), entre outubro de 2018 e fevereiro de 2020, nas unidades de saúde da ESF ou nos domicílios das participantes, conforme a disponibilidade delas. A coleta de dados ocorreu face a face, individualmente com cada gestante, com duração média de uma hora. Quanto à ordem de aplicação dos instrumentos, primeiramente aplicou-se um questionário estruturado para investigação das características sociodemográficas/econômicas e clínicas, e na sequência as escalas referentes ao AMF, apoio social, funcionalidade familiar, estresse percebido e sintomas depressivos.

Além do mais, previamente à coleta de dados, houve uma capacitação dos entrevistadores, bem como um estudo piloto com 36 gestantes cadastradas em uma unidade da ESF (que não foram incluídas nas análises do estudo), com a finalidade de padronizar os procedimentos de coleta de dados da pesquisa.

### Tratamento e análise dos dados

Foi adotada a análise multivariada por meio da MEE. Primeiramente, foi elaborado um modelo hipotético, no intuito de avaliar as inter-relações entre o AMF, considerado como desfecho principal, e as demais variáveis averiguadas: semanas gestacionais, aglomeração do domicílio, sintomas de depressão, estresse percebido, apoio social e funcionalidade familiar.

 Segundo o modelo hipotetizado, a idade gestacional (semanas de gestação) foi colocada em uma posição de correlação direta com o desfecho principal ^(^ 7 ^-^ 8 ^,^ 13 ^-^ 15 ^)^ . Portanto, teorizou-se que a aglomeração do domicílio também exerce efeitos diretos sobre o AMF, por ser um possível marcador da condição socioeconômica da gestante ^(^ 4 ^,^ 6 ^,^ 15 ^)^ . Similarmente, o estresse percebido apresenta relação com o AMF ^(^ 5 ^,^ 7 ^,^ 9 ^,^ 15 ^)^ , assim como os sintomas depressivos ^(^ 4 ^-^ 5 ^,^ 7 ^,^ 9 ^-^ 11 ^,^ 14 ^)^ . Já o apoio social possui efeitos diretos e indiretos sobre o AMF, em uma inter-relação mediada pelo estresse percebido e pelos sintomas de depressão ^(^ 4 ^-^ 6 ^,^ 12 ^,^ 15 ^)^ . A mesma suposição foi feita para o construto funcionalidade familiar ^(^ 7 ^,^ 14 ^)^ . 

 Além do mais, a Figura 1 ilustra as relações diretas e indiretas entre as variáveis investigadas no modelo proposto. As variáveis observadas estão representadas por retângulos e as correlações são indicadas por setas (da variável independente para a dependente) ^(^ 31 ^)^ . 

**Figura 1:** - Modelo hipotético para analisar os fatores inter-relacionados ao apego materno-fetal em gestantes assistidas na Atenção Primária à Saúde. Montes Claros, MG, Brasil, 2018-2020

 Na análise estatística dos dados, inicialmente, as variáveis categóricas foram descritas por meio de suas distribuições de frequências. Já as variáveis numéricas foram descritas por meio da média, do desvio-padrão (DP), dos valores mínimo e máximo, dos coeficientes de assimetria (sk) e curtose (ku). Valores de sk> 3 e ku> 10 foram acatados como indicadores de violação do pressuposto da normalidade ^(^ 31 ^)^ . Por outro lado, os valores perdidos ( *missing* ) foram imputados pela média. 

 Em seguida, foi ajustado o modelo multivariado por meio da MEE. Foram estimados efeitos diretos e indiretos representados por coeficientes padronizados (CPs), cujas significâncias estatísticas foram avaliadas pela razão crítica (RC), ao nível de 5%. Além disso, os CPs padronizados foram interpretados da seguinte maneira: com efeito pequeno, quando os valores eram próximos de 0,10; médio, quando próximos de 0,30; e grande, se superiores a 0,50 ^(^ 32 ^)^ . Os efeitos indiretos, intermediados por variáveis intermediárias, foram calculados multiplicando-se os coeficientes dos caminhos indiretos do modelo. 

 Para a avaliação da adequação do ajuste global do modelo foram apreciados os seguintes índices: razão do qui-quadrado pelos graus de liberdade (x ^2^ /gl), *confirmatory fit index* (CFI), *goodness of fit index* (GFI), *Tucker-Lewis index* (TLI) e *root mean square error of approximation* (RMSEA). O ajuste do modelo foi considerado satisfatório se x ^2^ /df ≤ 5,0; CFI, GFI e TLI ≥ 0,90; RMSEA < 0,10 ^(^ 33 ^)^ . 

 Já a organização e as análises estatísticas dos dados ocorreram no *software* IBM *Statistical Package for the Social Sciences* (SPSS) *Statistics* , versão 23.0. Em um momento anterior, os dados coletados foram submetidos a um controle de qualidade e dupla verificação. Além do mais, a modelagem estrutural foi processada no *software* *Analysis of Moment Structures* (IBM SPSS Amos 23.0). 

### Aspectos éticos

O presente trabalho acatou as regulamentações éticas internacionais e nacionais das pesquisas envolvendo seres humanos. O projeto foi aprovado pelo Comitê de Ética em Pesquisa Envolvendo Seres Humanos da Universidade Estadual de Montes Claros (CAAE 80957817.5.0000.5146, Parecer Consubstanciado número 2.483.623/2018 e Parecer Consubstanciado número 3.724.531/2019), e obteve concordância institucional da Secretaria Municipal de Saúde. As participantes com maioridade leram e assinaram o Termo de Consentimento Livre e Esclarecido. Contudo, as que possuíam menos de 18 anos apresentaram o Termo de Assentimento Livre e Esclarecido, além do Termo de Consentimento Livre e Esclarecido assinado por seus responsáveis legais.

## Resultados

 Um total de 1.279 gestantes participou da linha de base do estudo. No presente trabalho foram incluídos os dados de 937 entrevistadas, que se encontravam no segundo e terceiro trimestres gestacionais ( Figura 2 ). 

 Quanto às características sociodemográficas e clínicas das participantes da amostra analisada (n=937): 47,7% estavam na faixa etária de 21 a 30 anos, 77,2% referiram o estado conjugal com companheiro e 46,7% tinham renda familiar mensal de até 1.000 reais. Das entrevistadas: 55,0% estavam no 2º trimestre, 61,0% relataram gravidez não planejada, 48,7% eram nulíparas e 18,9% referiram aborto prévio. No que concerne aos escores do AMF, observou-se média de 92,6 (DP=±15,3) e o nível de apego médio foi o predominante, verificado em 575 (61,4%) gestantes. Além disso, os desvios da normalidade não foram muito severos para os coeficientes de assimetria e curtose das variáveis do modelo estrutural ( Tabela 1 ). 

**Figura 2:** - Fluxograma do processo de seleção da amostra de gestantes analisadas no estudo. Montes Claros, MG, Brasil, 2018-2020

**Tabela 1:** - Características das gestantes assistidas na Atenção Primária à Saúde e medidas descritivas das variáveis do modelo estrutural (n = 937). Montes Claros, MG, Brasil, 2018-2020

**Variáveis**		n*	**% [IC 95%** ^†^ **]**	
**Faixa etária (anos)**				
Até 20		203	22,4 [19,2-24,4]	
21 a 30		453	47,6 [45,3-51,6]	
Acima de 30		281	30,0 [27,1-33,0]	
**Situação conjugal**				
Com companheiro		721	77,2 [74,4-79,8]	
Sem companheiro		213	22,8 [20,2-25,6]	
**Cor autodeclarada**				
Parda		641	68,8 [65,7-71,7]	
Negra		147	15,8 [13,6-18,3]	
Branca		102	10,9 [9,1-13,1]	
Amarela		42	4,5 [3,4-6,0]	
**Escolaridade**				
Ensino fundamental		141	15,1 [12,8-17,4]	
Ensino médio		595	63,5 [60,4-55,6]	
Ensino superior		200	21,4 [18,8-24,0]	
**Renda familiar mensal (R$ ^‡^ )**				
Até 1.000,00		422	46,7 [43,4-50,0]	
1.000,00 a 2.000,00		280	31,0 [28,0-34,0]	
Acima de 2.000,00		202	22,3 [19,6-25,0]	
**Trimestre gestacional**				
2º		515	55,0 [51,8-58,2]	
3º		422	45,0 [41,8-48,2]	
**Planejamento da gestação**				
Sim		360	39,0 [359-42,1]	
Não		564	61,0 [57,9-64,1]	
**Aborto prévio**				
Não		742	81,1 [78,6-83,6]	
Sim		173	18,9 [16,4-21,4]	
**Apego materno-fetal**				
Baixo		10	1,1 [0,4-1,8]	
Médio		575	61,4 [59,3-64,5]	
Alto		352	37,5 [34,4-40,6]	
**Funcionalidade familiar**				
Família funcional		785	84,0 [81,6-86,4]	
Família disfuncional		149	16,0 [13,6-18,4]	
**Apoio social**				
Baixo		166	17,9 [15,4-20,4]	
Elevado		760	82,1 [79,6-84,6]	
**Sintomas depressivos**				
Ausentes/leves		560	61,3 [58,1-64,5]	
Moderados		142	15,5 [13,2-17,8]	
Graves		212	23,2 [20,5-25,9]	
**Estresse percebido**				
Ausência de estresse		768	83,1 [80,7-85,5]	
Presença de estresse		156	16,9 [14,5-18,3]	
**Variáveis do modelo estrutural**	**Média (DP** ^§^ **)**	**Min-Max** ^||^	**Sk** ^¶^	Ku**
Apego materno-fetal	92,6 (15,3)	29,0-120,0	-0,71	1,03
Funcionalidade familiar	8,5 (2,1)	0,0-10,0	-1,72	2,68
Apoio social	79,3 (18,3)	0,0-94,7	-1,39	1,52
Sintomas depressivos	15,3 (10,1)	0,0-57,0	1,17	1,22
Estresse percebido	23,5 (8,7)	1,0-56,0	0,15	0,57
Semanas gestacionais	26,3 (7,6)	13,6-45,1	0,11	-0,95
Aglomeração do domicílio	0,7 (0,3)	0,20-3,50	2,22	8,31

* n varia devido à ausência de informação ( *missing* )

^†^ IC 95% = Intervalo de 95% de confiança

^‡^ R$ = Real brasileiro (salário mínimo: R$ 954,00)

^§^ DP = Desvio-padrão

^||^ Min-Max = Mínimo e máximo

^¶^ sk = Assimetria

** ku = Curtose

 A Figura 3 apresenta as inter-relações entre os fatores investigados e os coeficientes estruturais padronizados. O modelo estrutural ajustado obteve indicadores de qualidade do ajuste adequados, com os seguintes valores: x ^2^ /gl=2,29, GFI=0,994, CFI=0,989, TLI=0,975, RMSEA=0,037 (IC 90%=0,016; 0,059, p-valor=0,823). De acordo com o modelo, as semanas de gestação exerceram efeito direto positivo sobre o desfecho AMF (β=0,29; p<0,001), enquanto a aglomeração do domicílio exibiu efeito direto negativo (β=-0,07; p=0,027). Inclusive, os sintomas depressivos tiveram efeito direto negativo sobre o desfecho (β=-0,11; p=0,003). Em contraste, o apoio social apresentou correlação positiva e direta com o apego (β=0,08; p<0,001), além de efeito indireto negativo intermediado pela variável sintomas depressivos (β=-0,29; p<0,001). Quanto à funcionalidade familiar, também se observou efeito direto positivo sobre o AMF (β=0,19; p<0,001), bem como efeito negativo em inter-relação com os sintomas depressivos (β=-0,20; p<0,001). 

 As magnitudes dos efeitos direto, indireto e total estão descritas na Tabela 2 . Na análise de mediação, apenas o estresse percebido não obteve evidência estatística de relação com o evento analisado. 

**Figura 3:** - Modelo estrutural ajustado dos fatores inter-relacionados ao apego materno-fetal em gestantes assistidas na Atenção Primária à Saúde (n = 937). Montes Claros, MG, Brasil, 2018-2020

**Tabela 2:** - Magnitudes dos efeitos direto, indireto e total estimados dos fatores inter-relacionados ao apego materno-fetal em gestantes assistidas na Atenção Primária à Saúde (n = 937). Montes Claros, MG, Brasil, 2018-2020

**Variável independente**	**Efeito**	**Variável dependente**	**Coeficiente padronizado**	**Total**	Valor de p*
	**Direto/Indireto**		**Direto/Indireto**	
Semanas gestacionais	Direto	Apego materno-fetal	0,29	0,29	<0,001
Aglomeração domiciliar	Direto	Apego materno-fetal	-0,07	-0,07	0,027
Sintomas depressivos	Direto	Apego materno-fetal	-0,11	-0,11	0,003
Estresse percebido	Direto	Apego materno-fetal	-0,02	-0,02	0,539
Apoio social	Direto	Apego materno-fetal	0,08		
	Indireto via sintomas depressivos		-0,29x(-0,11)=0,0319	0,12	<0,001
	Indireto via estresse percebido		-0,29x(-0,02)=0,0058		
	Direto	Sintomas depressivos	-0,29	-0,29	<0,001
	Direto	Estresse percebido	-0,29	-0,29	<0,001
Funcionalidade familiar	Direto	Apego materno-fetal	0,19		
	Indireto via sintomas depressivos		-0,20x(-0,11)=0,022	0,22	<0,001
	Indireto via estresse percebido		-0,17x(-0,02)=0,0034		
	Direto	Sintomas depressivos	-0,20	-0,20	<0,001
	Direto	Estresse percebido	-0,17	-0,17	<0,001

* Nível de significância estatística (p<0,05)

## Discussão

O presente estudo evidenciou as inter-relações entre AMF, idade gestacional, aglomeração domiciliar, sintomas depressivos, apoio social e funcionalidade familiar em gestantes brasileiras assistidas na APS. Por intermédio da MEE, foi possível compreender um sistema composto por múltiplos fatores e suas inter-relações.

 No modelo estrutural ajustado, foi observado que as semanas de gestação possuem relação direta com o evento pesquisado, demonstrando ser um resultado positivo. Resultado similar foi identificado em pesquisas com gestantes italianas ^(^ 8 ^)^ e brasileiras na região Sul do país ^(^ 13 ^-^ 14 ^)^ . Em vista disso, trata-se de um achado plausível, pois os comportamentos de AMF aumentam com o avanço da idade gestacional à medida em que a mulher sente os movimentos fetais, o que torna a experiência mais corporal para ela e acentua a interação mãe-bebê. A partir do quarto mês de gestação, com os movimentos do feto, a mãe passa a expressar de forma mais intensa representações, expectativas, preocupações e afetos em relação ao filho por nascer ^(^ 8 ^,^ 13 ^-^ 14 ^)^ . 

 Por outro lado, a aglomeração do domicílio apresentou relação negativa com o AMF nas gestantes entrevistadas: houve um comportamento de decréscimo dos escores de apego conforme o aumento da aglomeração de pessoas na residência. Esse fator pode ser considerado um marcador do *status* socioeconômico da mãe e de sua família que compromete a qualidade do vínculo afetivo mãe-feto. Pois ambientes domésticos positivos, estáveis e estimulantes possibilitam que a relação mãe-feto se estabeleça de forma mais saudável. Quando essa situação não é presente, pode influenciar negativamente a qualidade da interação materna, por constituir uma fonte de estresse familiar ^(^ 6 ^)^ . As baixas condições socioeconômicas e os contextos de vida desfavoráveis podem prejudicar a adaptação materna e familiar à gestação, uma vez que a gravidez requer uma reestruturação em várias dimensões ^(^ 3 ^)^ . 

 Outro aspecto negativo, neste inquérito epidemiológico, foi a presença de sintomas depressivos, que foram correlacionados negativamente ao AMF. Tal constatação tem sido evidenciada na literatura internacional, em países como Dinamarca ^(^ 4 ^,^ 34 ^)^ , China ^(^ 5 ^)^ , Irã ^(^ 11 ^)^ , Turquia ^(^ 9 ^)^ e Portugal ^(^ 10 ^)^ ; assim como em uma revisão sistemática ^(^ 7 ^)^ . No Brasil, foi igualmente observada em investigação de base domiciliar na região Sul ^(^ 14 ^)^ . A gestação pode não constituir um fator de proteção à saúde mental materna, ao contrário, pode situar a mulher em posição de vulnerabilidade ao sofrimento psíquico ^(^ 1 ^)^ . Acrescenta-se que as múltiplas mudanças vivenciadas no período gravídico exigem adaptações e podem favorecer o desenvolvimento de psicopatologias ^(^ 3 ^)^ , como a depressão, a qual é comum nessa fase ^(^ 7 ^)^ . Essa condição pode interferir no vínculo afetivo da gestante com o feto no pré-natal ^(^ 3 ^,^ 14 ^,^ 34 ^)^ . Além disso, teoriza-se que a depressão afeta a capacidade empática e a disponibilidade de afeto materno, então a gestante passa a considerar o feto como fonte de irritação ou culpa, resultando em um estado de distanciamento ^(^ 14 ^,^ 34 ^)^ . Todavia, não é possível atestar uma relação causal direta entre depressão e AMF, dada a natureza subjetiva e singular dessas condições, o que requer cautela na interpretação do resultado observado. Pesquisas qualitativas e longitudinais podem contribuir no aprofundamento do entendimento sobre tal constatação. 

 Paralelamente, as gestantes com sintomatologia depressiva tendem a experienciar a maternidade como sombria e permeada por sentimentos de culpa, tristeza, fadiga, baixa autoestima e desamparo. Pode acontecer a falta de confiança materna em desempenhar as atividades cotidianas e os cuidados com o futuro bebê conforme almejado, além de medo, tensão, pensamentos intrusivos de ameaça à vida ou ao bem-estar do filho. Logo, configura-se uma situação geradora de sofrimento e angústia para a mulher, que pode até mesmo ocultar seu estado mental com receio de ser estigmatizada. Há o impacto negativo na saúde materno-infantil, visto que os sintomas depressivos podem continuar no puerpério ^(^ 5 ^,^ 15 ^,^ 35 ^)^ . Sendo assim, filhos de mães deprimidas podem apresentar comprometimento no desenvolvimento afetivo, social e cognitivo ^(^ 1 ^)^ . Portanto, no âmbito da assistência de enfermagem na ESF, é recomendada a identificação precoce de mulheres em vulnerabilidade psicológica ^(^ 15 ^)^ , bem como a atenção voltada para a saúde mental das gestantes na assistência pré-natal, de modo a prevenir o quadro depressivo ^(^ 6 ^)^ . O cuidado humanizado também é necessário, conforme preconizado pelo Ministério da Saúde brasileiro em suas diretrizes oficiais ^(^ 17 ^-^ 18 ^,^ 36 ^-^ 37 ^)^ . 

 A assistência profissional ampliada, resolutiva e humanizada é importante ao se considerar que, nesta investigação, houve evidência estatística de relação positiva e direta entre o apoio social e o apego: o maior nível de apoio social repercutiu em maior intensidade de AMF. Além do mais, ocorreu efeito indireto negativo intermediado pela variável sintomas depressivos na inter-relação entre apoio social e o desfecho principal, isto é, quanto maior o apoio social da gestante, menor a intensidade dos sintomas de depressão, os quais impactaram negativamente no vínculo afetivo mãe-feto. A conexão entre apoio social e AMF também foi registrada em estudos realizados com gestantes dinamarquesas ^(^ 4 ^)^ , iranianas ^(^ 11 ^)^ e turcas ^(^ 9 ^)^ . O elevado apoio social pode contribuir para o bem-estar do binômio mãe-feto, pois as gestantes que têm uma rede de apoio e cuidados presumivelmente, ficam mais propensas ao autocuidado e ao vínculo afetivo com o feto ^(^ 14 ^)^ . Tal construto é importante para a manutenção da saúde mental, o enfrentamento de situações estressantes e a adequação de comportamentos maternos requeridos na fase gravídico-puerperal. Um bom apoio social pode ser um aspecto que protege contra a ocorrência de sintomas depressivos e promove a maternagem saudável. Já que o papel desse apoio é basilar para o amortecimento de fatores estressantes que ocorrem no cotidiano, principalmente no caso da gravidez, quando acontecem diversas modificações psicossociais e fisiológicas ^(^ 6 ^,^ 38 ^)^ . 

 No tocante à funcionalidade familiar, esta investigação evidenciou um resultado benéfico semelhante ao apoio social. Foi observado efeito direto positivo sobre o AMF, bem como efeito intermediado pelos sintomas depressivos. Inferiu-se que a adequada funcionalidade familiar implica em melhor AMF e em menor intensidade de sintomas depressivos, o que evidencia a repercussão favorável de uma rede familiar saudável sobre o evento em análise. De modo que, no período gestacional, a mulher precisa do apoio da família para poder se adaptar à novas condições oriundas da transição para a maternidade ^(^ 11 ^)^ . Essa base, especialmente em momentos de vulnerabilidade no ciclo vital, tem sido demonstrada como fator de proteção para a saúde mental materna ^(^ 1 ^,^ 14 ^)^ . A relação familiar satisfatória revela maior percepção de apoio prático e emocional, além de que fomenta sentimentos de pertencimento e potencializa o desempenho da função materna ^(^ 1 ^)^ . Isso contribui diretamente para um contexto de vida mais propício ao estabelecimento do AMF. No cenário comunitário, recomenda-se aos enfermeiros e às equipes da ESF responsáveis pela atenção pré-natal uma abordagem voltada para a promoção do bem-estar psicossocial e para as relações familiares saudáveis das gestantes ^(^ 15 ^)^ , segundo o estipulado na Política Nacional de Atenção Básica (PNAB) ^(^ 36 ^)^ e nas recomendações para o pré-natal de baixo risco ^(^ 37 ^)^ . 

 Face aos achados identificados neste estudo, informa-se que há um *corpus* de evidências internacionais ^(^ 4 ^-^ 5 ^,^ 9 ^,^ 11 ^,^ 34 ^)^ e brasileiras ^(^ 2 ^-^ 3 ^,^ 13 ^-^ 14 ^,^ 35 ^,^ 38 ^)^ que podem nortear a prática do enfermeiro da saúde da família. Tais evidências orientam o rastreio de gestantes usuárias dos serviços da APS em vulnerabilidade, com sintomas de depressão, dificuldade de adaptação à gravidez, pouco apoio social e familiar, que demandam cuidados direcionados à promoção da saúde mental. Logo, é necessário um maior suporte psicossocial e emocional às grávidas por meio de uma abordagem mais holística por parte das equipes da ESF, o que pode influenciar positivamente o AMF ^(^ 5 ^,^ 9 ^,^ 15 ^,^ 35 ^,^ 39 ^)^ . Além dos aspectos físicos, a dimensão subjetiva que permeia o período gestacional, como o AMF, deve ser considerada pelo enfermeiro e por demais profissionais de saúde da família, pois está associada a melhores resultados materno-infantis no período pós-parto. Além do mais, é preciso que tais profissionais avaliem o apego e o estimulem precocemente. Inclusive, os grupos de educação em saúde com as gestantes no pré-natal ofertado nas unidades da ESF podem ser benéficos, pois permitem às participantes o compartilhamento de medos, angústias e expectativas, propiciando a sensação de amparo ^(^ 2 ^,^ 39 ^)^ . 

 Tendo em vista a promoção do cuidado humanizado, resolutivo e integral, assim como a qualidade de vida do binômio materno-fetal, recomenda-se aos enfermeiros das equipes da ESF que a atenção pré-natal se baseie em diretrizes nacionais do Ministério da Saúde que direcionam a linha de cuidado materno-infantil. Pode-se destacar a PNAISM ^(^ 17 ^)^ , a Rede Cegonha ^(^ 18 ^)^ , a PNAB ^(^ 36 ^)^ e as orientações presentes no documento Cadernos de Atenção Básica sobre a prática profissional na atenção ao pré-natal de baixo risco ^(^ 37 ^)^ . No cotidiano da assistência em saúde, outra sugestão é a aplicação da EAMF nas consultas e nos grupos de pré-natal, a fim de identificar as gestantes que têm dificuldade em estabelecer um vínculo afetivo com o feto. Ademais, no âmbito da ESF e do SUS, é possível rastrear precocemente as gestantes e famílias que necessitem de abordagem profissional, com vistas à qualidade do vínculo mãe-feto ^(^ 15 ^)^ . 

Em conclusão, vale ainda evidenciar que este trabalho tem certas limitações. As informações foram autorrelatadas pelas participantes, logo estão propensas ao viés de aceitabilidade social e ao viés de memória. Embora o excesso de variáveis no modelo possa atrapalhar a qualidade do ajuste, outras variáveis, principalmente relativas ao perfil socioeconômico, ao religioso e ao estilo de vida não foram analisadas. Sugere-se a realização de outras pesquisas com desenho longitudinal prospectivo, para examinar a relação causal entre os fatores verificados e o AMF, com acréscimo das referidas variáveis. Outra recomendação é a inclusão de gestantes da área rural em estudos futuros.

A despeito dessas limitações, vale salientar aspectos positivos da presente investigação. Tratou-se de um amplo inquérito epidemiológico de base populacional, com amostra expressiva e abrangência de toda a zona urbana coberta pelas equipes da ESF. Foram aplicados instrumentos validados no Brasil, conferindo maior fidedignidade aos resultados. Além disso, a análise com MEE propiciou maior robustez e consistência à pesquisa. Em suma, os achados obtidos podem contribuir para agregar evidências epidemiológicas sobre a temática no contexto da APS, principalmente ao se reconhecer o ineditismo nacional deste inquérito, por envolver gestantes assistidas por equipes de saúde da família.

## Conclusão

Em resumo, o presente estudo evidenciou uma rede de inter-relações entre fatores preditores do AMF em gestantes assistidas por equipes da ESF. O modelo estrutural ajustado revelou que a maior idade gestacional, o maior apoio social e a melhor funcionalidade familiar tiveram uma relação positiva com o AMF. Em contrapartida, a maior aglomeração do domicílio e os sintomas depressivos implicaram negativamente nesse construto. Sinalizou-se um papel protetor do adequado apoio social e familiar à saúde mental das gestantes. Tais resultados são importantes para os enfermeiros da APS que realizam a assistência pré-natal, visto que indicam a necessidade de uma atenção ancorada na integralidade e na humanização, que propicie o bem-estar biopsicossocial, a saúde mental, a prevenção de sintomas depressivos e um AMF saudável.
